# Supplementary material for: Structural Basis for Receptor Activity-Modifying Protein-Dependent Selective Peptide Recognition by a G Protein-Coupled Receptor
Source: Mol Cell. 2015 Jun 18;58(6):1040–52. doi: 10.1016/j.molcel.2015.04.018 (PMC4504005; doi:10.1016/j.molcel.2015.04.018)
Supplement: Document S1. Supplemental Experimental Procedures, Figures S1 and S2, and Tables S1–S4 [file mmc1.pdf]

Molecular Cell

Supplemental Information

Structural Basis for Receptor Activity-Modifying Protein-Dependent Selective Peptide Recognition by a G Protein-Coupled Receptor

Jason M. Booe, Christopher Walker, James Barwell, Gabriel Kuteyi, John Simms, Muhammad A. Jamaluddin, Margaret L. Warner, Roslyn M. Bill, Paul W. Harris, Margaret A. Brimble, David R. Poyner, Debbie L. Hay, and Augen A. Pioszak

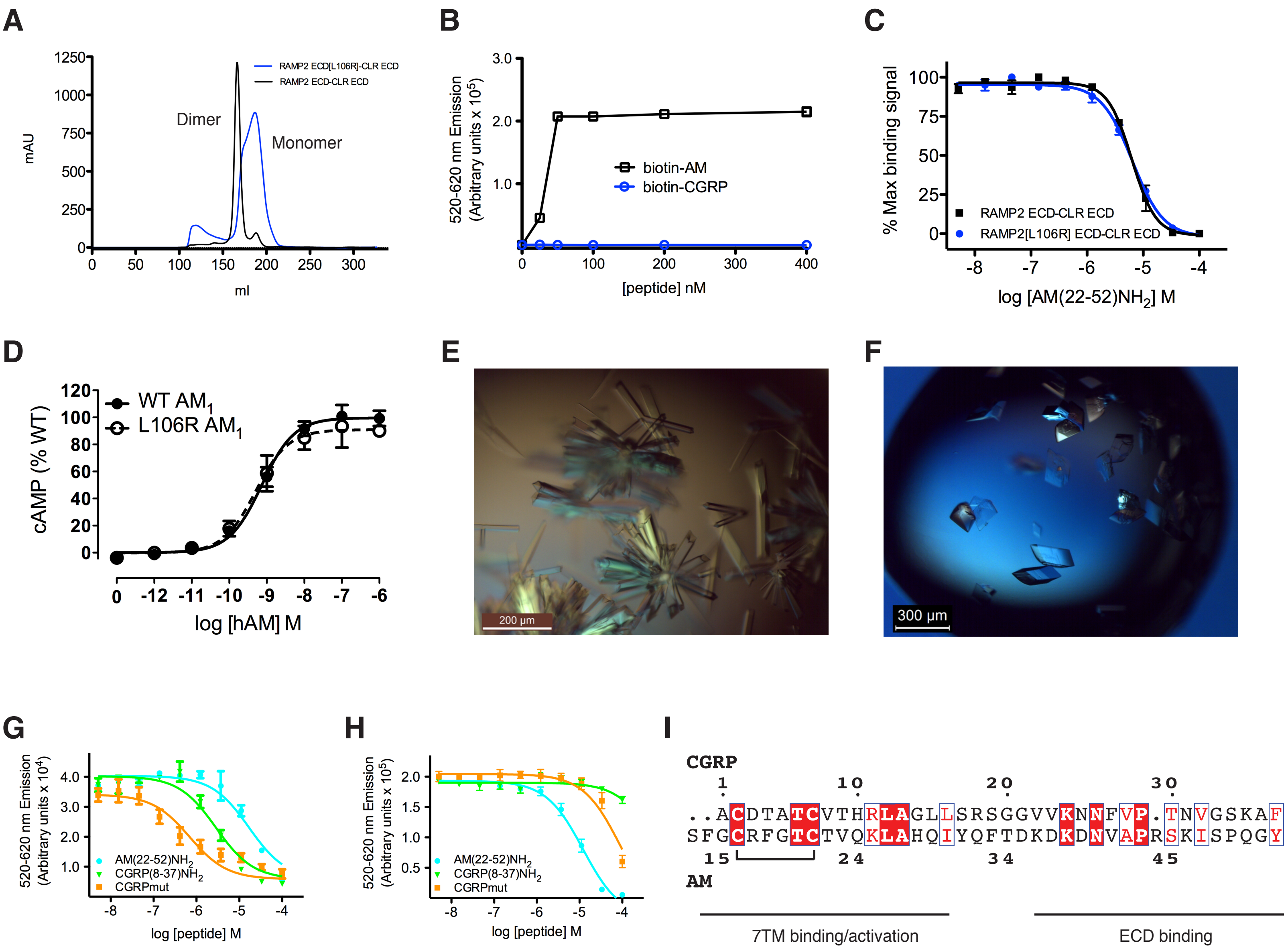

Supplemental Figure S1 (related to Figure 1). Protein characterization and crystallization. (A) Superdex200 HR gel-filtration elution profiles for MBP-RAMP2 ECD-$(GS)_5$-CLR ECD-H_6 and MBP-RAMP2 ECD [L106R]-$(GSA)_3$-CLR ECD-H_6 tethered fusion proteins. (B) AlphaScreen assay in saturation binding format for the RAMP2 [L106R] tethered fusion (150 nM) with the indicated concentrations of N-terminally biotinylated AM or CGRP. (C) Competition AlphaScreen assay for the indicated tethered fusion proteins (125 nM) and biotin-AM (125 nM) incubated with the indicated concentrations of AM(22-52)NH_2 competitor peptide. Data shown are representative of three independent experiments each performed in duplicate. The pIC_{50} values for the WT RAMP2 ECD-CLR ECD fusion and the RAMP2 [L106R]-CLR ECD fusion were 5.32 ± 0.08 and 5.27 ± 0.09, respectively. (D) Concentration-response curve for AM_1 receptor with the RAMP2 [L106R] mutant tested with hAM in a cAMP assay in COS-7 cells. (E) Crystals of the AM(25-52)NH_2-bound MBP-RAMP2 ECD [L106R]-$(GSA)_3$-CLR ECD-H_6 fusion. Individual rods were broken off the clusters for data collection. (F) Crystals of the CGRPmut-bound MBP-RAMP1 ECD-$(GSA)_3$-CLR ECD-H_6 fusion protein. (G) Competition AlphaScreen peptide binding assay for the MBP-RAMP1 ECD-$(GSA)_3$-CLR ECD-H_6 fusion protein (100 nM) and biotin-CGRP (100 nM) incubated with the indicated competitor peptides. The pIC_{50} values for AM(22-52)NH_2, CGRP(8-37)NH_2, and CGRPmut were 4.99 ± 0.13, 5.67 ± 0.10, 6.34 ± 0.09, respectively. (H) Competition AlphaScreen assay for the MBP-RAMP2 ECD [L106R]-$(GSA)_3$-CLR ECD-H_6 fusion protein (100 nM) and biotin-AM (100 nM) incubated with the indicated competitor peptides. The pIC_{50} value for AM(22-52)NH_2 was 4.89 ± 0.01. (I) Amino acid sequence alignment of human αCGRP and AM.

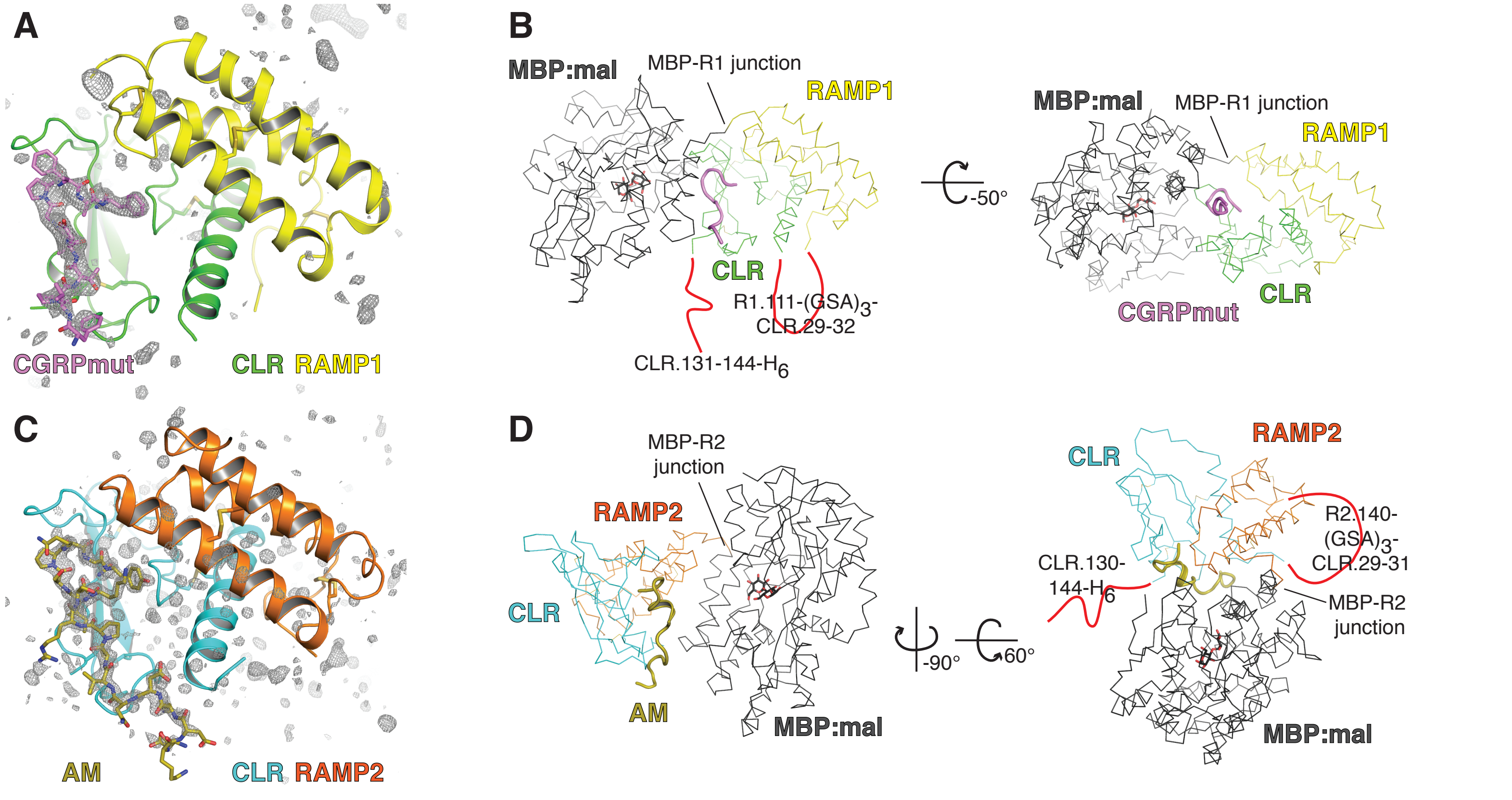

Supplemental Figure S2 (related to Figure 1). Electron density maps and structures of the MBP-tethered ECD fusion proteins. (A, C) The CGRP and AM_1 receptor ECD complex structures obtained after initial rebuilding of the molecular replacement models, but before addition of peptides or water molecules are shown with the mF_o-DF_c electron density maps (gray mesh) from these models contoured at 3σ. The CGRPmut and AM peptides from the final refined structures are included for reference. (B, D) Structures of the tethered fusion proteins highlighting the positions of MBP relative to the ECD complexes and disordered tethers and C-terminal regions (red lines). Molecule A is shown for the CGRP receptor structure. In the left image of panel B the CGRP receptor ECD complex is oriented the same as in panel A. In the left image of panel D the AM_1 receptor ECD complex is oriented the same as in Figure 1B, right image.

Table S1 (related to Fig 2). Summary of contacts between CGRP [D31, P34, F35] and CLR:RAMP1 ECD.

CGRP residue	Contact with CLR	Contact with RAMP1
F27	sc[2] VDW/HP[3] to D94sc	
T30	mc[1] N-H H-bond donate D94sc	
	mc C=O H-bond accept N128sc	
	sc H-bond donate D94sc	
	sc VDW/HP to W72sc, F92sc, F95sc	
V32	sc VDW/HP to F95sc, W72sc, Y124sc	
G33	mc C=O H-bond accept W121sc	
P34	mc C=O H-bond accept S117sc	
	sc VDW/HP to H114sc, A116sc	
F35	mc C=O H-bond accept R119sc	
	sc VDW/HP to S117sc	
F37	sc VDW/HP to W72sc, G71mc	sc VDW/HP to W84mc C=O and W84sc
		sc minor VDW/HP to P85sc
Amide	C=O H-bond accept T122mc N-H	
	NH_2 H-bond donate T122mc C=O	

[1]mc=main chain
[2]sc=side chain
[3]VDW/HP=van der Waals/hydrophobic contacts

Table S2 (related to Fig 2). Summary of contacts between AM and CLR:RAMP2 ECD.

AM residue	Contact with CLR	Contact with RAMP2
K38	mc[1] C=O H-bond accept Q93sc[2]	
D39	mc N-H H-bond donate T37sc	
	mc C=O H-bond accept Q93mc N-H	
N40	mc C=O H-bond accept D94mc N-H	
A42	mc N-H H-bond donate D94sc	
	sc VDW/HP[3] to F95sc, F92sc, W72sc	
P43	sc VDW/HP to F92sc, W72sc	
K46	sc VDW/HP to W72sc	sc H-bond donate E105sc
		sc H-bond donate E101sc
I47	sc VDW/HP to W72sc, F95sc, Y124sc	
S48	mc C=O H-bond accept W121sc	
P49	mc C=O H-bond accept S117sc	
	sc VDW/HP to H114sc, A116sc	
Q50	mc C=O H-bond accept R119sc	
Y52	sc VDW/HP to W72sc, G71mc	sc H-bond donate E101sc
		sc H-bond accept R97sc
		sc minor VDW/HP to F111mc/sc, P112sc
Amide	C=O H-bond accept T122mc N-H	
	NH_2 H-bond donate T122mc C=O	

[1]mc=main chain
[2]sc=side chain
[3]VDW/HP=van der Waals/hydrophobic contacts

Table S3 (related to Fig 4). Summary of receptor function when stimulated with hαCGRP (pEC_{50} and E_{max} values) and cell surface expression for mutants of CLR in the CGRP receptor.

	cAMP				Surface Expression
**Mutant**	Wildtype (pEC_{50})	Mutant (pEC_{50})	Fold[4] change	E_{MAX} (% WT)	HA-CLR (% WT)
T37A[1]	9.15 ± 0.17 (5)	10.08 ± 0.23* (5)	8.5 (↑)	129.1 ± 12.6	108.5 ± 8.6
W69A[2]	9.44 ± 0.18 (7)	<6 (7)	>1000	Undetectable (7)	84.0 ± 6.1 (7)
D70A[2]	9.55 ± 0.18 (7)	<6 (7)	>1000	Undetectable (7)	83.6 ± 5.1 (7)
W72A[2]	9.93 ± 0.19 (6)	8.29 ± 0.29* (6)	44	76.6 ± 3.6* (6)	96.0 ± 8.4 (7)
D90A[2]	10.00 ± 0.03 (4)	9.50 ± 0.10** (4)	3	116.2 ± 4.1 (4)	103.9 ± 3.5 (3)
Y91A[2]	9.94 ± 0.09 (4)	8.30 ± 0.17* (4)	44	101.8 ± 9.9 (4)	93.9 ± 2.1 (2)
F92A[2]	9.88 ± 0.10 (5)	8.28 ± 0.07** (5)	40	94.9 ± 9.2 (5)	103.5 ± 1.1 (3)
Q93A[3]	9.33 ± 0.07 (4)	9.34 ± 0.07 (4)	-	112.9 ± 24.8 (4)	108.7 ± 3.7(4)
D94A[2]	9.80 ± 0.10 (5)	<6 (5)	>1000	Undetectable (5)	107.8 ± 7.6 (3)
F95A[2]	9.86 ± 0.17 (5)	7.59 ± 0.29* (5)	186	69.8 ± 8.6* (5)	111.4 ± 2.3 (3)
K103A[2]	9.91 ± 0.14 (4)	8.11 ± 0.19* (4)	63	97.6 ± 11.1 (4)	109.5 ± 3.7 (3)
H114A[3]	9.44 ± 0.09 (6)	8.12 ± 0.13 (6)***	21	85.8 ± 10.4 (6)	109.3 ± 17 (4)
S117A[3]	9.34 ± 0.06 (3)	8.92 ± 0.14 (3)*	3	90.4 ± 22.8 (3)	104.5 ± 7.3 (4)
R119A[2]	9.91 ± 0.14 (4)	8.16 ± 0.20* (4)	56	95.0 ± 6.8 (4)	107.1 ± 6.8 (6)
W121A[3]	9.56 ± 0.12 (3)	<6 (3)	>1000	Undetectable (3)	98.9 ± 10 (4)
T122A[3]	9.23 ± 0.06 (10)	7.93 ± 0.10 (10)***	20	103.0 ± 14.9 (10)	105.4 ± 4.3 (4)
Y124A[2]	9.76 ± 0.09 (5)	7.85 ± 0.09** (5)	81	79.6 ± 2.5* (5)	112.9 ± 4.6 (3)

[1]Data taken from Mapping interaction sites within the N-terminus of the calcitonin gene-related peptide receptor; the role of residues 23-60 of the calcitonin receptor-like receptor. Barwell J, Miller PS, Donnelly D, Poyner DR. Peptides. 2010 Jan;31(1):170-6

[2]These experiments were conducted using the ^{3}H cAMP method. An additional set of experiments using two mutants with the AlphaScreen cAMP method as per the AM_1 receptor gave mean pEC_{50}s of WT 9.57 ± 0.11 (3) and D94A <6 (3); WT 9.03 ± 0.06 (6) and W72A 7.03 ± 0.14*** (6).

[3]These experiments were conducted using the AlphaScreen cAMP method as per the AM_1 receptor. In a fourth experiment, W121A gave a pEC_{50} of 8.07 with a WT pEC_{50} of 9.36.

[4]Unless otherwise stated, values are fold-decreases.

For cAMP (pEC_{50}) data are the combined mean ± SEM (individual experiments). * $p < 0.05$; ** $p < 0.01$; *** $p < 0.001$ versus wild type receptor by unpaired t-test. For E_{MAX} and surface expression data are the combined mean ± SEM (individual experiments). * $p < 0.05$; ** $p < 0.01$; versus wild type receptor by one-way ANOVA, followed by Dunnett's post-hoc test.

Table S4 (related to Fig 5). Summary of receptor function when stimulated with hAM (pEC_{50} and E_{max} values) and cell surface expression for mutants of CLR or RAMP2 in the AM_1 receptor.

	cAMP				Surface Expression
**Mutant**	Wildtype (pEC_{50})	Mutant (pEC_{50})	Fold change	E_{MAX} (% WT)	HA-CLR (% WT)
**Calcitonin receptor-like receptor (CLR)**					
T37A	9.08±0.11 (3)	8.58±0.21 (3)	3	96.5±5.6 (3)	87.3±13.6 (4)
W72A	9.23±0.12 (4)	7.13±0.12 (4)***	126	35.5±11.4 (4)***	86.4±10.2 (4)
F92A	9.24±0.09 (3)	7.58±0.21 (3)**	46	42.8±2.1 (3)**	96.9±12.9 (4)
Q93A	9.25±0.10 (3)	9.20±0.12 (3)	-	111.7±14.5 (3)	99.5±2.7 (4)
D94A	9.13±0.15 (3)	8.20±0.14 (3)*	9	78.3±14.0 (3)	98.4±23.2 (4)
F95A	9.15±0.10 (3)	7.40±0.32 (3)**	56	73.7±6.2 (3)	111.8±18.4 (4)
H114A	9.07±0.03 (3)	8.28±0.25 (3)*	6	64.0±12.2 (3)	90.9±19.5 (4)
S117A	9.18±0.12 (3)	9.09±0.19 (3)	-	80.5±5.3 (3)	99.4±26.9 (4)
R119A	9.26±0.08 (4)	8.70±0.10 (4)**	4	84.9±8.3 (4)	102.5±24.3 (4)
W121A	9.32±0.10 (3)	6.99±0.05 (3)***	214	30.4±1.5 (3)***	102.2±15.3 (4)
T122A	9.31±0.18 (3)	8.41±0.15 (3)*	8	49.4±5.2 (4)**	106.0±15.7 (4)
Y124A	9.44±0.12 (3)	<6	>1000	Undetectable (3)	62.9±7.67 (4)**
**Receptor activity-modifying protein 2 (RAMP2)**					
L106R	9.13±0.15 (3)	9.28±0.20 (3)	-	91.4±7.9 (3)	121.7±24.9 (4)
R97A	9.25±0.10 (3)	9.16±0.11 (3)	-	82.9±1.0 (3)	85.0±5.2 (4)
E101A	9.26±0.09 (6)	7.84±0.09 (6)***	26	90.4±9.37 (6)	100.8±4.39 (8)
E105A	9.05±0.07 (3)	9.08±0.09 (3)	-	91.7±6.5 (3)	103.1±4.3 (4)
E101A/ R97A	9.44±0.12 (3)	8.07±0.03 (3)***	23	50.4±8.90 (3)	85.7±7.56 (4)
E101A/ E105A	9.44±0.12 (3)	7.70±0.06 (3)***	55	46.3±4.90 (3)	75.3±8.32 (4)

For cAMP (pEC_{50}) data are the combined mean ± SEM (individual experiments). * $p < 0.05$; ** $p < 0.01$; *** $p < 0.001$ versus wild type receptor by unpaired t-test. For E_{MAX} and surface expression data are the combined mean ± SEM (individual experiments). ** $p < 0.01$; *** $p < 0.001$ versus wild type receptor by one-way ANOVA, followed by Dunnett's post-hoc test.

Supplemental Experimental Procedures

Plasmid construction and protein production and characterization. Bacterial pETDuet1 expression plasmids encoding tethered MBP-hRAMP1.24-111-(Gly-Ser-Ala)$_3$-hCLR.29-144-H$_6$ and MBP-hRAMP2.55-140-(Gly-Ser-Ala)$_3$-hCLR.29-144-H$_6$ (amino acid numbers indicated) fusion proteins co-expressed with DsbC were constructed using the Gibson Assembly cloning method with Gibson Assembly master mix (New England Biolabs). Plasmids for expression of tagged full-length CLR and RAMP1 or -2 receptors in mammalian cells were previously described (Barwell et al., 2010; Watkins et al., 2014). Site directed mutagenesis was performed with the QuikChange II kit (Agilent) or using the Gibson Assembly method. Primer sequences are available upon request. All constructs were verified by automated DNA sequencing.

The tethered ECD fusion proteins were expressed in *E. coli*, purified, and characterized for peptide binding with an AlphaScreen peptide binding assay as previously described (Moad and Pioszak, 2013), except that the AlphaScreen competition assays also included 0.3% (v/v) Triton X-100 in the reaction buffer. Triton X-100 minimized, but did not completely prevent, apparent aggregation of the CGRP(27-37)NH_2 [D31, P34, F35] peptide that was surprisingly observed only with the MBP-hRAMP1.24-111-(Gly-Ser-Ala)$_3$-hCLR.29-144-H$_6$ protein and not with MBP-hRAMP1.24-111-(Gly-Ser)$_5$-hCLR.29-144-H$_6$ or MBP-hRAMP2.55-140 [L106R]-(Gly-Ser-Ala)$_3$-hCLR.29-144-H$_6$ proteins (data not shown). The apparent aggregation of the CGRP analog prevented it from fully competing the binding signal to background levels and hence the reported IC_{50} values for the CGRP analog peptides binding to the CGRP receptor crystallization construct are likely a bit higher than the true values. Competitor

peptide concentrations higher than 200 μM were avoided because some of the peptides began to exhibit non-specific inhibition at concentrations > 200 μM as assessed in control reactions in which the donor and acceptor beads were brought together by a Biotin-$(Gly)_6$-$(His)_6$ peptide. The binding experiments were conducted at least three times with each independent experiment performed with duplicate samples. pIC_{50} values are stated as the mean of the replicate independent experiments ± S.E.M. Although slight variation in pIC_{50} values for a given peptide in assays conducted on different days was occasionally observed, the rank order of IC_{50} values for the various peptides and the magnitude of their differences were very reproducible.

Peptides. Custom synthetic peptides for binding studies and crystallization were from RS Synthesis (Louisville, Kentucky) other than the CGRP(27-37)NH_2 [D31, P34, F35] peptide used for crystallization, which was assembled by Fmoc SPPS on Rink amide polystyrene resin using a Tribute synthesizer (Protein Technologies, Tucson, Az) with 20% (v/v) piperidine in DMF as Fmoc deblocking reagent (2 x 5 mins) and HATU/DIPEA (20 mins) as coupling reagents. The peptide was cleaved from the resin with concomitant removal of side chain protecting groups with 95% TFA/2.5% TIPS/2.5% water (v/v/v) for 2 h and recovered by precipitation into cold diethyl ether and isolated by centrifugation (221 mg). Purification of a portion (110 mg) by RP-HPLC on a C18 column (Waters Xterra, 19 x 300 mm) afforded the title compound (27.4 mg, >95% purity by HPLC), observed mass (ESI+) $(M+H)^{1+}$ = 1195.0, calculated mass 1196.4. For cell-based assays Human AM(1-52) was from Bachem and hαCGRP(1-37) was synthesized in-house (PWH) or was from Bachem.

Crystallization, data collection, structure determination, and analysis. The tethered MBP-RAMP1 ECD-CLR ECD and MBP-RAMP2 ECD [L106R]-CLR ECD fusion proteins were incubated for 1 h on ice in the presence of CGRP(27-37)NH_2 [D31, P34, F35] or AM(25-52)NH_2 (1:1.3 protein:peptide molar ratio), respectively, in 10 mM Tris-HCl, pH 7.5, 50 mM NaCl, 1 mM EDTA, 1mM maltose and spin concentrated to 30 mg/ml for crystallization. Crystals were grown by the hanging drop vapor diffusion method at 20 °C with a reservoir solution of 22% PEG3350, 8% Tacsimate (Hampton Research), pH 6.0 for the CGRP receptor complex or 19% PEG3350, 0.1 M Tris-HCl, pH 8.3, 225 mM sodium acetate, and 20% ethylene glycol for the AM_1 receptor complex. Microseeding was used to obtain the best CGRP receptor complex crystals for data collection. CGRP receptor complex crystals were cryoprotected by dialysis to mother liquor solution containing 12% PEG400; AM_1 receptor complex crystals were suitably cryoprotected in their growth condition. Crystals were flash frozen in liquid nitrogen and diffraction data were collected remotely at beamline 21-ID-G (λ = 0.97857 Å) of the Advanced Photon Source (Argonne, IL). Data from single crystals were indexed, integrated, and scaled with HKL2000 v. 705b (Otwinowski and Minor, 1997) and further processed/analyzed with the CCP4 suite v.6.4.0 (Winn et al., 2011) in preparation for molecular replacement (MR). The structures were solved with Phaser v. 2.5.6 (McCoy et al., 2007) using an MBP search model with maltose removed (PDB 3C4M) followed by ligand-free CLR:RAMP1 ECD (PDB 3N7S) or CLR:RAMP2 ECD heterodimer search models (PDB 3AQF). The MR solutions were rigid body refined with REFMAC5 v. 5.8.0073 (Murshudov et al., 1997) treating MBP, CLR, and RAMP1 or RAMP2 as separate rigid bodies. At this stage $2mF_o\text{-}DF_c$ and $mF_o\text{-}DF_c$ electron density maps clearly

showed bound maltose and CGRPmut or AM peptide. The models were completed by iterative rounds of manual rebuilding in COOT (Emsley et al., 2010) and TLS and restrained refinement with REFMAC5. NCS restraints were applied to the three molecules in the ASU of the CGRP receptor complex structure with the restraints relaxed for areas where the molecules differed. Structure analysis used PyMol (Schrodinger) and programs in the CCP4 suite and figures were prepared with PyMol. Structural superpositions were performed with the PyMol align command (for Cα atoms) utilizing outlier rejection.

Homology Modeling. Homology models of RAMP3 were generated using Modeller 9v12 (Sali and Blundell, 1993). CGRPmut-bound CLR:RAMP1 and AM-bound CLR:RAMP2 template structures were used either singularly or in combination to generate the models. 6000 models were generated, refined using Rosetta 3.5 (Rohl et al., 2004) and ranked using the OPUS_PSP scoring function (Lu et al., 2008). The 600 best scoring structures were then clustered into 0.1nm bins using the g_cluster function as implemented in Gromacs (Pronk et al., 2013). The best scoring structure from the largest, best scoring cluster was then selected.

Supplemental References

Barwell, J., Miller, P.S., Donnelly, D., and Poyner, D.R. (2010). Mapping interaction sites within the N-terminus of the calcitonin gene-related peptide receptor; the role of residues 23-60 of the calcitonin receptor-like receptor. Peptides *31*, 170-176.

Emsley, P., Lohkamp, B., Scott, W.G., and Cowtan, K. (2010). Features and development of Coot. Acta Crystallogr D Biol Crystallogr *66*, 486-501.

Lu, M., Dousis, A.D., and Ma, J. (2008). OPUS-PSP: an orientation-dependent statistical all-atom potential derived from side-chain packing. J Mol Biol *376*, 288-301.

McCoy, A.J., Grosse-Kunstleve, R.W., Adams, P.D., Winn, M.D., Storoni, L.C., and Read, R.J. (2007). Phaser crystallographic software. J Appl Crystallogr *40*, 658-674.

Moad, H.E., and Pioszak, A.A. (2013). Selective CGRP and adrenomedullin peptide binding by tethered RAMP-calcitonin receptor-like receptor extracellular domain fusion proteins. Protein Sci *22*, 1775-1785.

Murshudov, G.N., Vagin, A.A., and Dodson, E.J. (1997). Refinement of macromolecular structures by the maximum-likelihood method. Acta Crystallogr D Biol Crystallogr *53*, 240-255.

Otwinowski, Z., and Minor, W. (1997). Processing of X-ray diffraction data collected in oscillation mode. In Methods in Enzymology, C.W.J. Carter, and R.M. Sweet, eds. (New York: Academic Press), pp. 307-326.

Pronk, S., Pall, S., Schulz, R., Larsson, P., Bjelkmar, P., Apostolov, R., Shirts, M.R., Smith, J.C., Kasson, P.M., van der Spoel, D*., et al.* (2013). GROMACS 4.5: a high-throughput and highly parallel open source molecular simulation toolkit. Bioinformatics *29*, 845-854.

Rohl, C.A., Strauss, C.E., Misura, K.M., and Baker, D. (2004). Protein structure prediction using Rosetta. Methods Enzymol *383*, 66-93.

Sali, A., and Blundell, T.L. (1993). Comparative protein modelling by satisfaction of spatial restraints. J Mol Biol *234*, 779-815.

Watkins, H.A., Walker, C.S., Ly, K.N., Bailey, R.J., Barwell, J., Poyner, D.R., and Hay, D.L. (2014). Receptor activity-modifying protein-dependent effects of mutations in the calcitonin receptor-like receptor: implications for adrenomedullin and calcitonin gene-related peptide pharmacology. Br J Pharmacol *171*, 772-788.

Winn, M.D., Ballard, C.C., Cowtan, K.D., Dodson, E.J., Emsley, P., Evans, P.R., Keegan, R.M., Krissinel, E.B., Leslie, A.G., McCoy, A*., et al.* (2011). Overview of the CCP4 suite and current developments. Acta Crystallogr D Biol Crystallogr *67*, 235-242.
