# Supplementary material for: Structural Basis for Receptor Activity-Modifying Protein-Dependent Selective Peptide Recognition by a G Protein-Coupled Receptor
Source: Mol Cell. 2015 Jun 18;58(6):1040–52. doi: 10.1016/j.molcel.2015.04.018 (PMC4504005; doi:10.1016/j.molcel.2015.04.018)
Supplement: Document S2. Article plus Supplemental Information [file mmc2.pdf]

Molecular Cell

Article

Structural Basis for Receptor Activity-Modifying Protein-Dependent Selective Peptide Recognition by a G Protein-Coupled Receptor

Graphical Abstract

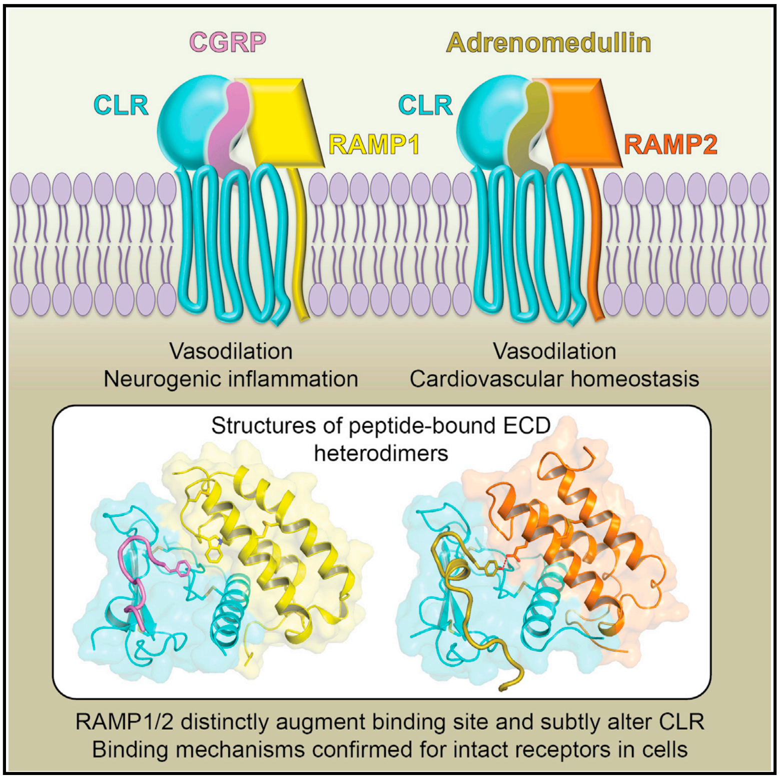

Authors

Jason M. Booe, Christopher S. Walker, James Barwell, ..., David R. Poyner, Debbie L. Hay, Augen A. Pioszak

Correspondence

augen-pioszak@ouhsc.edu

In Brief

Booe et al. report two crystal structures that reveal how selectivity of the GPCR CLR for CGRP and AM peptides is modulated by RAMP proteins. The peptides similarly occupy a shared binding site on CLR. RAMPs augment the binding site with distinct contacts to the peptides and subtly alter CLR.

Highlights

- Crystal structures reveal how CGRP and AM peptides bind their heterodimeric receptors
- CGRP and AM occupy a shared binding site on CLR with minimal contact to the RAMPs
- Peptide binding modes were confirmed for intact receptors in cells by mutagenesis
- Peptide selectivity arises from RAMP-specific contacts and subtle alteration of CLR

Accession Numbers

4RWF
4RWG

Booe et al., 2015, Molecular Cell *58*, 1040–1052
June 18, 2015 ©2015 The Authors
http://dx.doi.org/10.1016/j.molcel.2015.04.018

CellPress

Molecular Cell
Article

Structural Basis for Receptor Activity-Modifying Protein-Dependent Selective Peptide Recognition by a G Protein-Coupled Receptor

Jason M. Booe,[1] Christopher S. Walker,[2] James Barwell,[3,5] Gabriel Kuteyi,[3,6] John Simms,[3] Muhammad A. Jamaluddin,[2] Margaret L. Warner,[1] Roslyn M. Bill,[3] Paul W. Harris,[4] Margaret A. Brimble,[4] David R. Poyner,[3] Debbie L. Hay,[2] and Augen A. Pioszak[1,*]
[1]Department of Biochemistry and Molecular Biology, University of Oklahoma Health Sciences Center, Oklahoma City, OK 73104, USA
[2]School of Biological Sciences and Maurice Wilkins Centre, University of Auckland, Auckland 1142, New Zealand
[3]School of Life and Health Sciences, Aston University, Birmingham, B4 7ET, UK
[4]School of Chemical Sciences and Maurice Wilkins Centre, University of Auckland, Auckland 1142, New Zealand
[5]Present address: Adaptimmune Limited, 91 Park Drive, Milton Park, Abingdon, OX14 4RY, UK
[6]Present address: Department of Biochemistry, University of Oxford, Oxford, OX1 3QU, UK
*Correspondence: augen-pioszak@ouhsc.edu
http://dx.doi.org/10.1016/j.molcel.2015.04.018
This is an open access article under the CC BY license (http://creativecommons.org/licenses/by/4.0/).

SUMMARY

Association of receptor activity-modifying proteins (RAMP1-3) with the G protein-coupled receptor (GPCR) calcitonin receptor-like receptor (CLR) enables selective recognition of the peptides calcitonin gene-related peptide (CGRP) and adrenomedullin (AM) that have diverse functions in the cardiovascular and lymphatic systems. How peptides selectively bind GPCR:RAMP complexes is unknown. We report crystal structures of CGRP analog-bound CLR:RAMP1 and AM-bound CLR:RAMP2 extracellular domain heterodimers at 2.5 and 1.8 Å resolutions, respectively. The peptides similarly occupy a shared binding site on CLR with conformations characterized by a β-turn structure near their C termini rather than the α-helical structure common to peptides that bind related GPCRs. The RAMPs augment the binding site with distinct contacts to the variable C-terminal peptide residues and elicit subtly different CLR conformations. The structures and accompanying pharmacology data reveal how a class of accessory membrane proteins modulate ligand binding of a GPCR and may inform drug development targeting CLR:RAMP complexes.

INTRODUCTION

G protein-coupled receptors (GPCRs) are a large family of cell surface receptors that regulate a multitude of biological processes in response to a diverse array of stimuli and they are important drug targets. The class B/Secretin family GPCRs in humans include 15 receptors that are activated by diverse neuropeptides, peptide paracrine factors, and peptide endocrine hormones (Hoare, 2005). These receptors are less well understood than the larger class A/Rhodopsin family, despite their physiological and clinical importance. Class B GPCRs comprise an extracellular domain (ECD) of about 120 amino acids in addition to the 7-transmembrane (7TM) domain in the membrane. The ECD has an N-terminal α-helix and a set of β sheets held together by three disulfide bonds (Archbold et al., 2011). Peptides bind class B GPCRs via a "two-domain" model whereby their C-terminal region binds the ECD and their N-terminal region binds and activates the 7TM domain. Crystal structures are available for class B GPCR ECDs with bound peptides related to PTH, CRF, GIP, and GLP-1 (Pal et al., 2010; Parthier et al., 2007; Pioszak et al., 2008, 2009; Pioszak and Xu, 2008; Runge et al., 2008; Underwood et al., 2010) and a consensus has emerged from these studies. The peptides bind as extended α helices to the same region of the receptor, in a groove between the N and C termini of the isolated ECDs. For PTH, GIP, and GLP-1 families, the peptides are closest to the N terminus; for the CRF-related peptides, they are displaced to be closer to the C terminus.

Although this model of binding is valid for several class B GPCRs, it cannot apply to all class B receptors. In particular, there are problems understanding the binding of members of the calcitonin (CT) family of peptides; calcitonin gene-related peptides alpha and beta (αCGRP, βCGRP), adrenomedullin (AM), adrenomedullin 2/intermedin (AM2), amylin (Amy), and CT (Hong et al., 2012; Poyner et al., 2002). These C terminally amidated peptides have a range of actions including neurogenic inflammation (CGRP), vasodilation/cardioprotection (CGRP, AM, and AM2), and regulation of blood and lymphatic vascular development (AM), nutrient intake and blood glucose (Amy), and bone turnover (CT). CGRP antagonists showed promise for the treatment of migraine and AM may be of value for the treatment of cardiovascular disorders (Durham and Vause, 2010; Karpinich et al., 2011). An Amy analog is used to treat insulin-dependent diabetes patients (Edelman et al., 2008) and CT has been long used to treat bone disorders (Purdue et al., 2002).

CGRP, AM, and AM2 binding to their cognate class B receptor, the calcitonin receptor-like receptor (CLR), is dependent

on association of CLR with one of three accessory membrane proteins that determine ligand selectivity; receptor activity-modifying proteins (RAMPs) 1, 2, or 3 (Hong et al., 2012; McLatchie et al., 1998). RAMPs have an ECD of about 100 amino acids and a single TM segment (Parameswaran and Spielman, 2006). CLR:RAMP1 is a CGRP receptor, CLR:RAMP2 preferentially recognizes AM and is called the AM_1 receptor, and CLR:RAMP3 binds both AM and AM2 with high affinities and is called the AM_2 receptor. Amy by itself has a low affinity for the class B CT receptor (CTR); however, when CTR associates with any of the RAMPs, its affinity for Amy is markedly increased (Christopoulos et al., 1999; Poyner et al., 2002). CTR alone is the receptor for CT. Thus, the RAMPs profoundly alter the behavior of CLR and CTR. Although RAMPs are best characterized for their effects on CLR/CTR, they also interact with several other class B GPCRs and with certain class A/Rhodopsin and class C/Glutamate family GPCRs, making it particularly important to understand the molecular basis for RAMP actions (Bouschet et al., 2005; Lenhart et al., 2013; Wootten et al., 2010). RAMPs provide an excellent opportunity to explore how accessory membrane proteins can modulate GPCR pharmacology.

Crystal structures are available for ligand-free and small molecule antagonist-bound CLR:RAMP1 and ligand-free CLR:RAMP2 ECD complexes, but these provide little insight into how peptides bind or how RAMPs determine selectivity (Kusano et al., 2012; ter Haar et al., 2010). Extensive mutagenesis on the RAMPs (Qi and Hay, 2010) only provided clear evidence for the involvement of one RAMP residue (RAMP1 W84) in the binding of CGRP and two residues (RAMP2 F111 and E101) for AM binding (Moore et al., 2010; Watkins et al., 2014). It has not been possible to interpret these data mechanistically. A further complication is that it appears unlikely that CGRP and AM bind as extended helices as seen with other class B peptide ligands; there is evidence that only a small portion of these peptides form α helices and that at their C termini, there are one or more turn structures (Breeze et al., 1991; Carpenter et al., 2001; Pérez-Castells et al., 2012; Watkins et al., 2013b). Consequently, the mechanism of RAMP action and the mode of binding of CT family peptides remain unknown. Here, we describe high-resolution crystal structures of CGRP analog-bound CLR:RAMP1 and AM-bound CLR:RAMP2 ECD heterodimers that reveal bound peptide conformations starkly different from other class B GPCR peptide ligands, explain how RAMPs determine peptide selectivity, and provide molecular templates to guide drug development targeting CLR:RAMP complexes.

RESULTS

Engineering CLR:RAMP ECD Complexes for Crystallization

We previously reported a tethered fusion protein approach to engineer the CLR:RAMP1 and CLR:RAMP2 ECD complexes for crystallization (Moad and Pioszak, 2013), inspired by previous successes using maltose binding protein (MBP) as a "crystallization module" for class B GPCR ECDs (Kumar et al., 2011; Pal et al., 2010; Pioszak et al., 2008, 2009, 2010; Pioszak and Xu, 2008). MBP-RAMP1 or MBP-RAMP2 ECD-CLR ECD fusion proteins in which the two ECDs were covalently tethered with a flexible $(Gly\text{-}Ser)_5$ linker were designed to ensure complex stability and enforce 1:1 CLR:RAMP stoichiometry. The tethered RAMP1-CLR ECD fusion was a monomer, whereas the tethered RAMP2-CLR ECD fusion purified as a dimer, but the physiological relevance of oligomerization is unknown. Both proteins selectively bound their respective peptides but failed to yield crystals in the presence of peptides.

We reasoned that tether flexibility and oligomerization of the AM_1 receptor ECD complex hindered the crystallization efforts. We produced new constructs with a $(Gly\text{-}Ser\text{-}Ala)_3$ tether designed to decrease flexibility and we identified a single amino acid substitution in the RAMP2 ECD, L106R, which prevented dimerization of the tethered RAMP2-CLR fusion protein (Figure S1A) by disrupting a putative oligomerization interface identified by examining crystal packing in the ligand-free CLR:RAMP2 ECD structure (Kusano et al., 2012). The monomeric RAMP2 L106R-tethered construct retained selectivity for AM over CGRP and bound $AM(22\text{-}52)NH_2$ essentially identical to the wild-type tethered fusion in an AlphaScreen competition binding assay (IC_{50} ~5–15 μM) (Figures S1B, S1C, and S1H). In a cell-based cAMP signaling assay the full-length AM_1 receptor with RAMP2 [L106R] exhibited wild-type response to AM (Figure S1D; Table S4). High-quality crystals of MBP-RAMP2 ECD [L106R]-$(GSA)_3$-CLR ECD grown in the presence of $AM(25\text{-}52)NH_2$ were readily obtained (Figure S1E). Crystals of MBP-RAMP1 ECD-$(GSA)_3$-CLR ECD grown in the presence of $CGRP(20\text{-}37)NH_2$ diffracted poorly (data not shown); fortunately, high-quality crystals were obtained in the presence of a high-affinity CGRP analog $CGRP(27\text{-}37)NH_2$ [D31, P34, F35] (Rist et al., 1998) (Figure S1F). In the competition assay, the CGRP receptor crystallization construct was selective for CGRP over AM and bound the CGRP analog with higher affinity (IC_{50} ~0.46 μM) than $CGRP(8\text{-}37)NH_2$ (IC_{50} ~2 μM) (Figure S1G). The CGRP analog also bound the AM_1 receptor crystallization construct with higher affinity than wild-type CGRP but was still lower affinity than AM (Figure S1H). The crystallized proteins thus exhibited peptide selectivity consistent with the intact receptors. The peptides in both crystal forms are antagonist fragments that lack the N-terminal 7TM domain-activating region (Figure S1I). The CGRP analog will hereafter be referred to as CGRPmut.

Structures of the CGRPmut-Bound CLR:RAMP1 and AM-Bound CLR:RAMP2 ECD Heterodimers

Diffraction data for the CGRPmut- and AM-bound receptor complexes were collected to resolutions of 2.5 and 1.8 Å, respectively (Table 1). The structures were solved by molecular replacement (MR) and refined to good R_{work} and R_{free} values (Table 1). Three copies of the tethered CGRP receptor fusion and one copy of the tethered AM_1 receptor fusion were present in the asymmetric units. Molecule A (Mol A) of the CGRPmut-bound structure had the best electron density and lowest B-factors (Table 1); unless otherwise noted the figures use Mol A. The peptide-bound structures are shown in Figures 1A and 1B. The $mF_o\text{-}DF_c$ electron density maps for the rebuilt MR models showed clear, unambiguous density for CGRPmut and AM (Figures S2A and S2C). MBP sits over the bound peptides, but it does not appear to alter their binding (Figures S2B and S2D).

Table 1. Data Collection and Refinement Statistics

	MBP-RAMP1-CLR:CGRP(27-37) NH_2 [D31, P34, F35]	MBP-RAMP2-CLR:AM(25-52)NH_2
Data collection		
Space group	C2	$P2_12_12_1$
Cell dimensions		
a, *b*, *c*	172.81 Å, 104.62 Å, 136.48 Å	71.45 Å, 84.28 Å, 115.76 Å
α, β, γ	90°, 122.43°, 90°	90°, 90°, 90°
Resolution	50.0–2.45 Å (2.49–2.45 Å)[a]	50.0–1.76 Å (1.79–1.76 Å)
R_{merge}	0.058 (0.647)	0.068 (0.966)
$CC_{1/2}$	(0.858)	(0.525)
$I / \sigma I$	24.30 (1.76)	30.13 (1.05)
Completeness	99.8% (98.6%)	99.9% (98.2%)
Redundancy	4.2 (3.9)	7.2 (5.0)
Refinement		
Resolution (Å)	50.0-2.45 Å	50.0-1.76 Å
No. reflections	72,185	66,505
R_{work} / R_{free}	0.200/0.243	0.157/0.200
Protein molecules/ ASU	3	1
No. atoms (Mol 1/2/3)		
MBP	2,885/2,865/2,859	2,898
RAMP1 or RAMP2	705/657/705	721
CLR	805/981/744	805
CGRP or AM	86/86/75	148
Water	70	394
B-factors (Mol 1/2/3)		
MBP	71.62/94.58/96.57	37.14
RAMP1 or RAMP2	72.26/101.20/96.41	30.93
CLR	61.60/67.61/88.84	35.75
CGRP or AM	72.24/101.71/81.79	44.55
Water	52.85	39.32
RMS deviations		
Bond lengths	0.013 Å	0.020 Å
Bond angles	1.516°	1.929°
Ramachandran Analysis[b]		
Preferred regions	95.72%	96.8%
Allowed regions	4.04%	3.2%
Outliers	0.24%	0%

[a]Values in parentheses are for highest-resolution shell.
[b]As defined in COOT.

The final models include CGRPmut residues 27–37 and AM residues 35–52 (residues 25–34 were disordered) and the majority of the tethered fusion proteins other than the tethers and ~20 residues at the C terminus, which were disordered (Figures S2B and S2D). The tethers appeared to be longer than necessary, making it unlikely that they altered RAMP-CLR interactions.

CGRPmut and AM occupy similar positions near CLR loops 2, 3, and 4; only their C termini are in proximity to the RAMPs (Figures 1A and 1B). Strikingly, CGRPmut adopts a receptor-bound conformation devoid of secondary structure. Receptor-bound AM lacks secondary structure other than one α-helical turn. Shared turn structures near the peptide C termini similarly position the C-terminal residues adjacent to α2 and the α2-α3 loop of the RAMPs. CGRPmut and AM occupy the same face of the CLR ECD as observed for other class B GPCRs with their positions more similar to that of CRF than PTH (Figure 1C). Helix-breaking Pro residues are prevalent in the CGRP, AM, and AM2 sequences and the four residue segment prior to the C-terminal residue contains turn-favoring Pro or Gly residues, consistent with the observed peptide conformations (Figure 1D). The structures are consistent with our knowledge of the architectures of the intact receptor complexes. The ECDs are oriented such that their C termini could continue toward the membrane with a similar number of residues between the termini visible in the structures and the predicted start of the TM segments (~17 residues for CLR and ~8 for the RAMPs). The peptides are oriented such that their N termini containing the receptor-activating regions would be directed toward the 7TM domain.

CGRPmut and AM primarily contact CLR, but key RAMP contacts are also formed (Tables S1 and S2). Two key features of the peptide-binding sites are a hydrophobic patch extending from the base of CLR loop 4 to loop 3 and a pocket extending from the base of CLR loop 4 to loop 2 and the RAMPs (Figure 2). The CLR W72 bulge, previously called the “Trp shelf” (ter Haar et al., 2010), demarcates patch and pocket. The patch comprises the Trp shelf, F92, F95, and Y124. The pockets comprise the Trp shelf, D70, G71, W121, T122, Y124, and RAMP1 W84 and P85 in the CGRP receptor (Figures 2A and 2B) or RAMP2 R97, E101, E105, and P112 in the AM_1 receptor (Figures 2C and 2D). CGRPmut G33-A36 and AM S48-G51 form type II β-turns that contact CLR loop 4 in part via hydrogen bonds between the turn main chain and CLR S117, R119, and W121 side chains (Figures 2B and 2D). The β-turns enable the peptide C termini to occupy their respective pockets where their amide groups hydrogen bond with the CLR T122 main chain and their C-terminal residues pack against the Trp shelf, CLR G71, and RAMP1 W84 from the α2-α3 loop, which makes hydrophobic contact with CGRPmut F37 (Figures 2A and 2B), or RAMP2 R97, E101, and E105 from α2, which hydrogen bond with AM Y52 and K46 (Figures 2C and 2D). Prior to the β-turns CGRPmut V32 and AM I47 similarly contact the patch, but moving backward thence the peptides diverge in their interactions. CGRPmut T30 forms main chain- and side chain-mediated hydrogen bonds with CLR D94 on loop 3 and contacts the patch via its side chain methyl group (Figures 2A and 2B). The single helical turn in AM enables K46 to contact the Trp shelf and pack against AM Y52 and AM P43 and A42 contact the patch (Figures 2C and 2D). AM K38-A42 form a series of main chain-mediated hydrogen bonds with the main chain of CLR loop 3 and the side chains of D94 in loop 3 and T37 on α1 (Figure 2D). For the CGRPmut-bound structure, 94% of the solvent accessible surface area (ASA) of the ECD complex buried at the interface with the peptide is from CLR (478 $Å^2$) and only 6% is from RAMP1 (29 $Å^2$). More ASA is buried at the interface with AM, but the majority is still from CLR, 90% (781 $Å^2$), whereas RAMP2 contributes only 10% (85 $Å^2$).

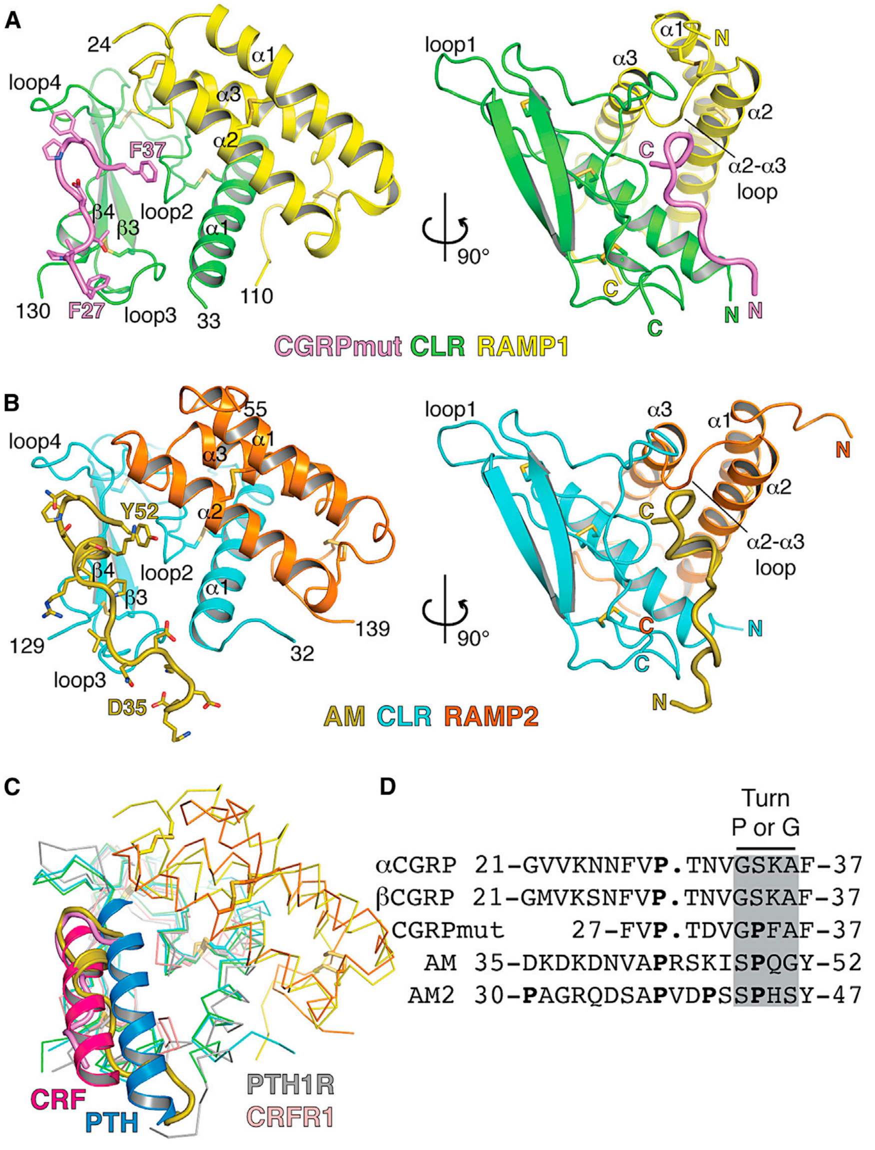

Figure 1. Peptide-Bound CGRP and AM$_1$ Receptor ECD Heterodimer Structures

(A and B) CGRPmut- and AM-bound complexes in cartoon representation with disulfide bonds as sticks and secondary structure elements labeled. Peptide, CLR, and RAMP terminal residues are numbered. Peptide side chains are shown as sticks in the left images but are omitted in the right images. MBP is not shown. The color scheme is consistent throughout the figures with carbon atoms varied in color to distinguish the proteins/peptides and oxygen, nitrogen, and sulfur atoms in CPK colors.

(C) Superpositions of the peptide-bound complexes with the PTH:PTH1R ECD (PDB: 3C4M) and CRF:CRFR1 ECD (PDB: 3EHU) structures. The receptors are shown as Cα traces and the peptides as cartoons.

(D) Amino acid sequences of the C-terminal regions of the human CGRP, CGRPmut, AM, and AM2 peptides. The turn structure region is highlighted in gray and Pro residues are in bold. See also Figures S1 and S2.

Comparisons to Ligand-free and Small Molecule Antagonist-Bound Structures

Superpositions of CGRPmut-bound and ligand-free CLR:RAMP1 complexes (ter Haar et al., 2010) revealed clamp-like movement of CLR loops 3 and 4 upon CGRPmut binding, presumably mediated by the CGRPmut T30-CLR D94 interaction and β-turn contacts with CLR loop 4 including the CGRPmut F35-CLR S117 interaction (Figures 3A and 3B). CLR R119 shifts to accommodate the peptide and RAMP1 F83 rotates away from CLR loop 4 (Figure 3B). The RAMP1 position relative to CLR varies in the structures (Figure 3A), but the positions of the α2-α3 loop and W84, which contacts CGRPmut F37, remain relatively similar (Figures 3A and 3B).

The CGRPmut-bound structure explains antagonism by the CGRP receptor-selective small molecule drugs olcegepant and telcagepant. Superposition of the CGRPmut-bound and drug-bound structures (ter Haar et al., 2010) indicated that olcegepant and telcagepant block key interactions of the CGRP C-terminal amide and F37 with the receptor pocket by hydrogen bonding with the CLR T122 main chain at the base of the pocket and packing of their piperidyl moieties against the Trp shelf and G71 (Figures 3C and 3D). Olcegepant also hydrogen bonds with CLR D94, thereby blocking the CGRP T30-CLR D94 interaction (Figure 3C). The position of RAMP1 relative to CLR in the peptide-bound versus drug-bound structures varies such that the drugs appear to favor RAMP1 α2 shifting closer to the small molecule binding sites (Figure 3E), presumably due to drug-RAMP1 interactions including packing against W74 (Figures 3C and 3D).

Superposition of the AM-bound and ligand-free CLR:RAMP2 complexes (Kusano et al., 2012) revealed minor CLR conformational differences involving the N-terminal region of α1 moving toward AM in the AM-bound state presumably due to AM-CLR T37 contacts (Figure 3F). CLR loops 3 and 4 do not move as in the CGRPmut-bound structure and there are no significant side-chain conformational differences at the peptide-binding site. The position of RAMP2 relative to CLR varies in the two structures (Figure 3F), but the ligand-free RAMP2 position is probably constrained by formation of the dimer of heterodimers in which the C-terminal end of RAMP2 α2 occupies the peptide-binding site of CLR from the opposing heterodimer (Figure 3G). Dimerization of the CLR:RAMP2 ECD heterodimer thus occludes the AM-binding site, which suggested that the RAMP2 L106R substitution (at the end of RAMP2 α2) was key to obtaining AM-bound crystals.

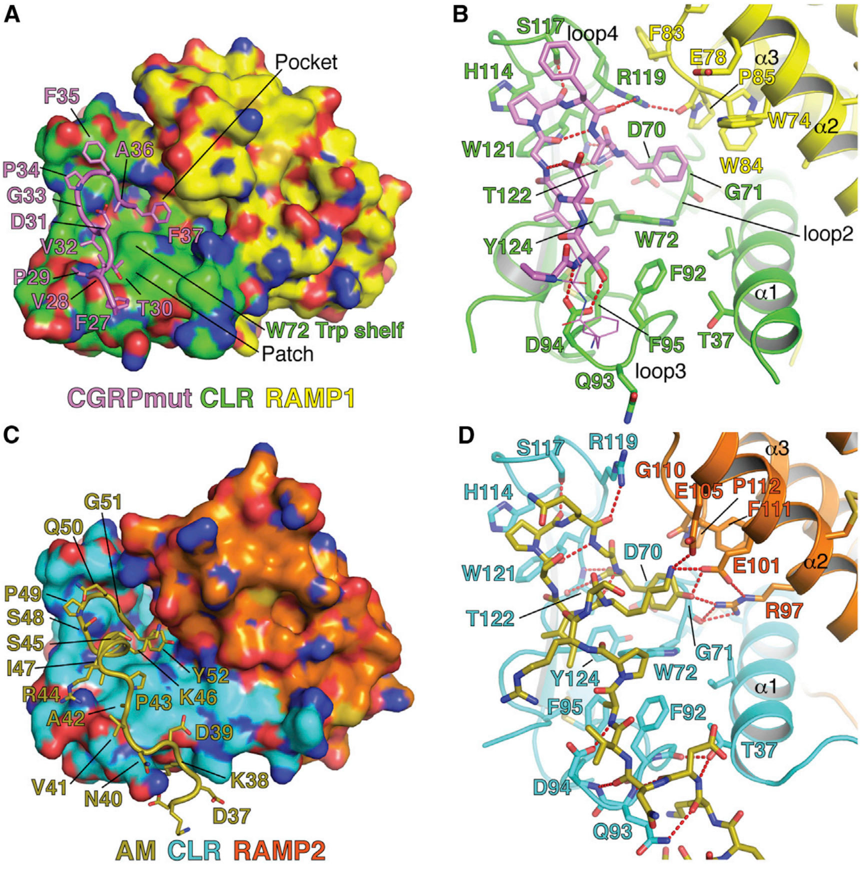

Figure 2. CGRPmut and AM Interactions with Their Receptor ECD Complexes

(A and C) The peptide-bound structures viewed with the receptor complexes in molecular surface representation and the peptides as cartoons with side chains as sticks.

(B and D) Detailed views with the receptors in cartoon representation and selected receptor residues and the peptides as sticks. Red dashes are hydrogen bonds. Peptide residues are labeled in (A) and (C) and receptor residues are labeled in (B) and (D). In (B), CGRPmut residues F27 and V28 are shown as lines for visualization of the CGRPmut T30-CLR D94 interaction. See also Tables S1 and S2.

Validation of the Structures for Intact CGRP and AM_1 Receptors in Cells

To validate the structures, we constructed several Ala substitution mutants in the CLR and RAMP2 ECDs, and they were analyzed for their effects on peptide-stimulated cAMP formation in COS-7 cells. For the CGRP receptor, Ala substitution of CLR W69, D70, K103, or Y91 significantly reduced CGRP potency, likely due to the structural roles of these residues (Figure 4, Table S3). Ala substitution of CLR W72, F92, D94, F95, H114, R119, W121, T122, or Y124, which are contacted by CGRPmut in the structure, reduced potency of the full-length CGRP >20-fold with the CLR D94A mutant being strikingly defective.

For the AM_1 receptor, Ala substitution of CLR W72, F92, F95, W121, or Y124, which are contacted by AM in the structure, resulted in >40-fold decreases in AM potency (Figure 5A, Table S4). CLR D94, H114, R119, or T122 mutants were less deleterious with 4- to 9-fold decreases in AM potency. Mutation of RAMP2 E101 yielded 26-fold reduced AM potency (Table S4; Watkins et al., 2014). Surprisingly, Ala substitution of RAMP2 R97 or E105 did not affect AM signaling potency despite their contacts with AM (Figure 5B). RAMP2 R97A/E101A and E101A/E105A double mutants had defects similar to the E101A single mutant. These data emphasize that RAMP2 E101 provides the crucial contacts to AM. The Ala substitutions did not significantly alter receptor cell surface expression levels other than reduced AM_1 receptor expression with CLR Y124A (Tables S3 and S4).

Peptide Selectivity Determinants

RAMPs may confer selectivity by providing distinct contacts to the peptides, altering CLR conformation, or a combination of the two. Superposition of the CGRPmut- and AM-bound structures indicated that the RAMPs augment the peptide-binding site pocket with distinct residues from their α2-α3 loop and α2 (Figure 6A). RAMP1 W84 in the α2-α3 loop makes hydrophobic contact with the CGRPmut F37 phenyl ring. This contact would be lost in RAMP2, which has the smaller F111 at the equivalent position. RAMP2 E101 on α2 hydrogen bonds with AM K46 and Y52. The equivalent RAMP1 W74 cannot make these contacts. Two other peptide-proximal RAMP positions differ: RAMP1 F83/RAMP2 G110 on the α2-α3 loop and RAMP1 A70/RAMP2 R97 on α2 (Figure 6B). The F83/G110 position is close to CLR loop 4 and the R119 side chain that has different conformations in the two structures. RAMP2 R97, which participates in the hydrogen bond network near AM Y52, would sterically clash with a Trp at position 111. The small A70 in RAMP1 avoids a clash with W84.

The positions of RAMP1 and RAMP2 relative to CLR differ in the two structures and the RAMPs elicit subtly different CLR conformations (Figure 6A). Equivalent RAMP1/2 Cα positions at the end of α2 differ by ~3 to 4.5 Å such that RAMP2 α2 is closer to the peptide-binding site than RAMP1 α2. A similar 3 to 4.5 Å displacement of the RAMP1/2 α3 helices is accompanied by shifts in the position of the C-terminal end of CLR α1 (Figures 6A and 6B). The RAMPs and CLR α1 appear to move somewhat as a unit relative to the remainder of CLR, which is also evident in the comparisons of the peptide-bound structures to the ligand-free and small molecule antagonist-bound structures (Figures 3A, 3E, and 3F). The subtly different CLR loop 2 positions in the structures may reflect RAMP-dependent differences at the interface with CLR α1 propagated to loop 2 via CLR W69 (Figures 6A and 6B).

To explore the contribution of RAMP binding site augmentation to selectivity, we constructed RAMP "swap" mutants in which the four variable residue positions near the peptide C termini were reciprocally exchanged between RAMP1 and

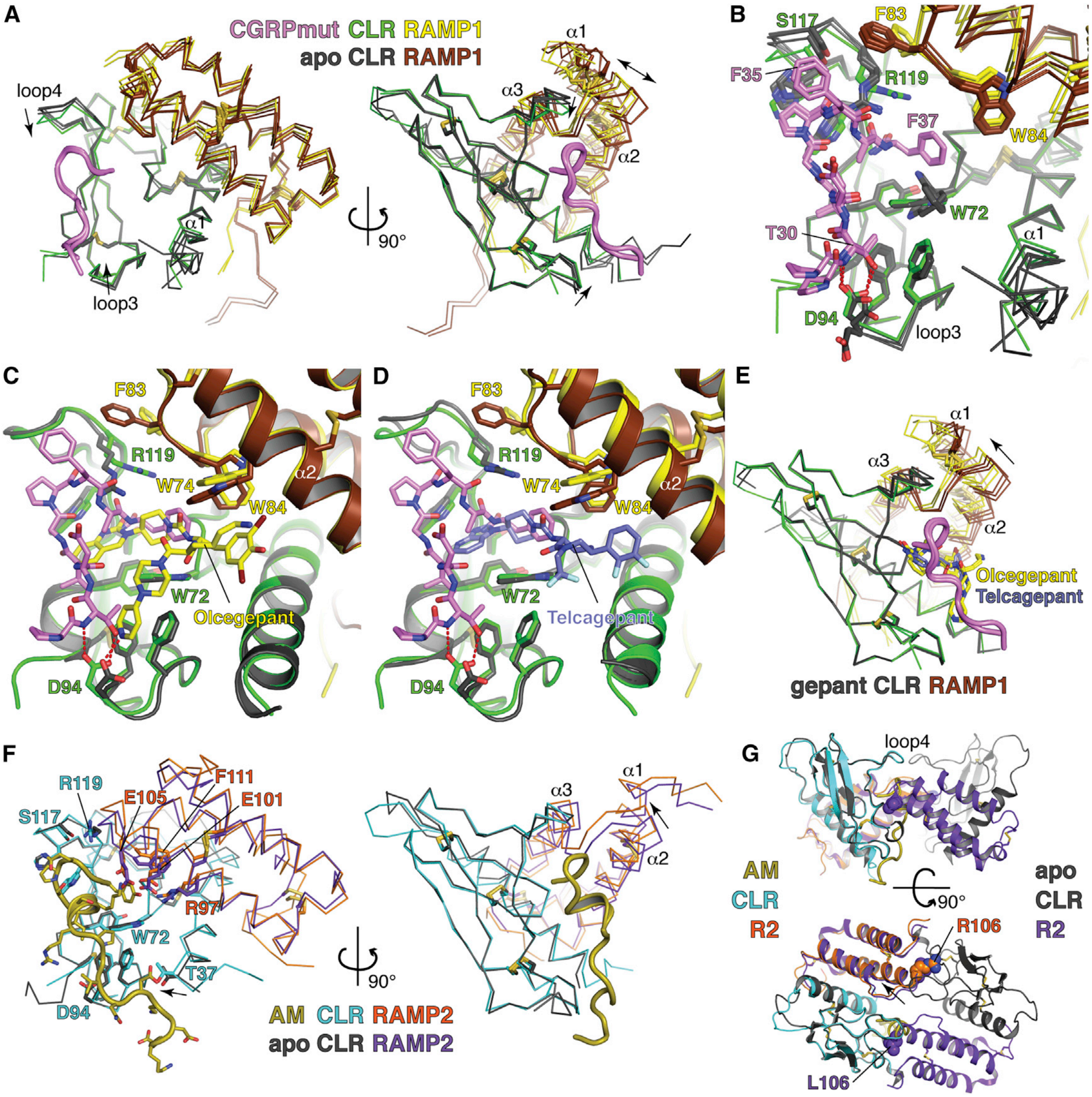

Figure 3. Comparisons of the CGRPmut- and AM-Bound Structures to Ligand-free and Small Molecule Antagonist-Bound Structures

(A) CGRPmut-bound complexes from Mol A and Mol C were aligned with four independent ligand-free CLR:RAMP1 complexes (PDB: 3N7P) based on the CLR positions. Mol B of the CGRPmut-bound structure was omitted because crystal packing altered its conformation. Receptors are shown as Cα traces. Arrows denote the directions of loop movements upon CGRPmut binding. The double-headed arrow highlights variability in the RAMP1 position relative to CLR.

(B) Detailed view of differences between the CGRPmut-bound and ligand-free states of the CLR:RAMP1 ECD complex. CGRPmut F27 and V28 are omitted for clarity.

(C and D) Superposition of CGRPmut-bound and olcegepant-bound (PDB: 3N7S) (C) or telcagepant-bound (PDB: 3N7R) (D) structures aligned based on the CLR positions. The peptide and small molecules are shown as sticks.

(E) Superpositions of the CGRPmut-bound structures with two independent olcegepant-bound complexes and a single telcagepant-bound complex based on the CLR positions. The arrow indicates the direction of movement of RAMP1 from the small molecule antagonist-bound to CGRPmut-bound states.

(F) Superposition of the AM-bound and ligand-free (PDB: 3AQF) CLR:RAMP2 ECD structures aligned based on the CLR positions. Receptors are shown as Cα traces and selected receptor and peptide residues as sticks. Arrows indicate directions of movement from ligand-free to AM-bound states.

(G) Putative dimer of ligand-free CLR:RAMP2 ECD heterodimers with the AM-bound CLR:RAMP2 ECD [L106R] heterodimer superimposed based on the CLR positions. The receptors and peptide are in cartoon representation and residues L/R106 are in space-filling representation. The arrow indicates the shift of RAMP2 in the AM-bound structure as compared to the ligand-free state. The top image is oriented similar to that in (F), right image.

RAMP2 (A70/R97, W74/E101, F83/G110, and W84/F111) and we tested their response to CGRP and AM in the cAMP assay (Figures 6C and 6D). The clearest effect was one of a modest decrease in cognate ligand potency. Accordingly, CGRP potency decreased ~10-fold at the CGRP receptor that included the RAMP1 mutant with RAMP2 residues and AM potency decreased ~50-fold in the AM_1 receptor with the RAMP2 mutant that contained RAMP1 residues. Thus, swapping these RAMP

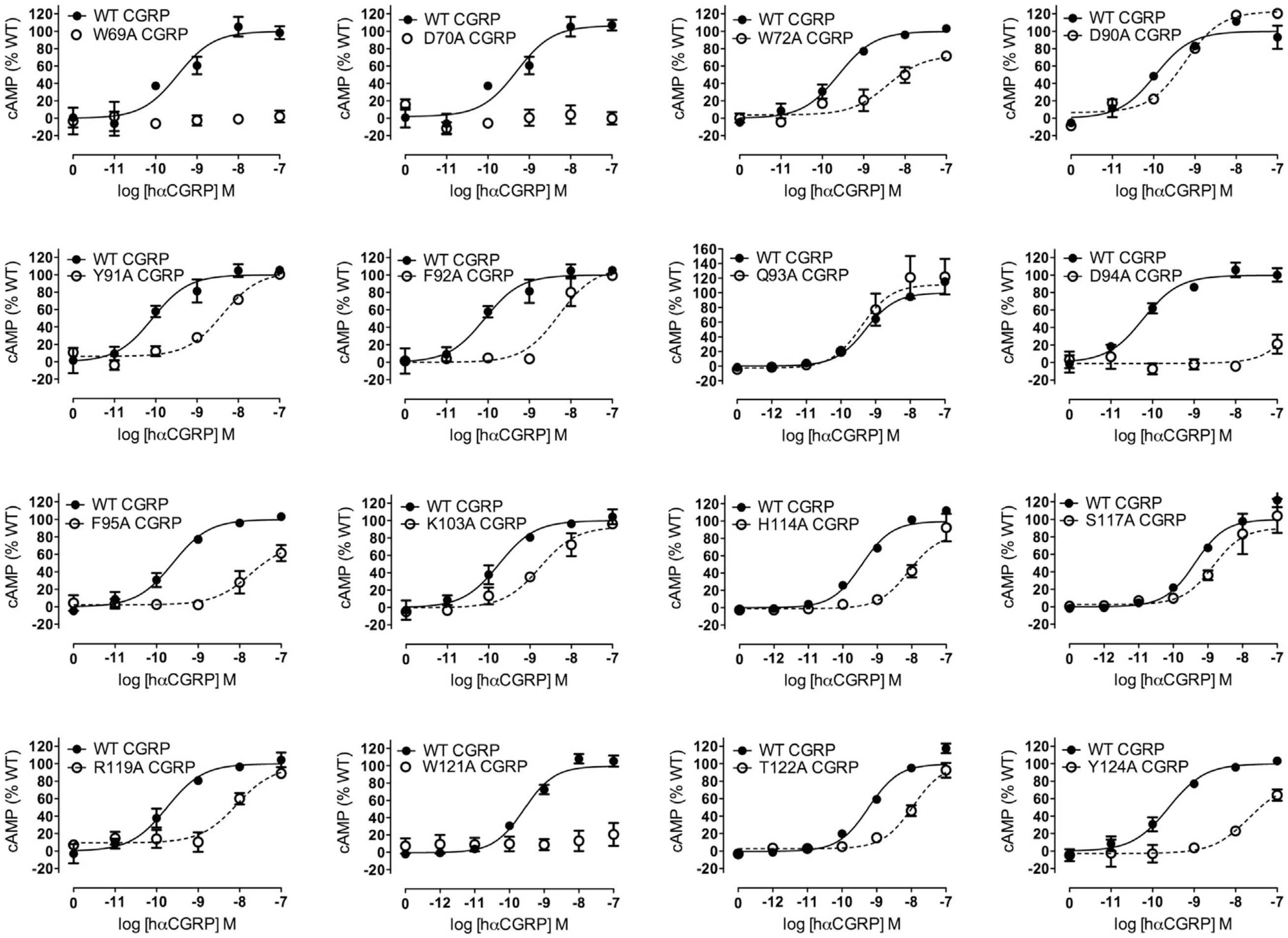

Figure 4. Validation of the CGRPmut-Bound ECD Heterodimer Structure for the Intact CGRP Receptor Transiently Expressed in COS-7 Cells
Concentration-response curves for each of the CLR alanine substitution mutants tested with hαCGRP in cAMP signaling assays. Data are represented as mean ± SEM. See also Table S3.

residues was insufficient to exchange pharmacological profiles, but the differing RAMP1/2 positions probably complicated the experiment.

We turned to peptide swap experiments to test whether reciprocal exchanges of the C-terminal residues of minimal ECD complex-binding CGRP and AM peptides (Moad and Pioszak, 2013) could exchange their receptor selectivity. In the competition AlphaScreen assay, CGRP(27-37)NH_2 [F37Y] retained the ability to bind the CGRP receptor ECD complex (Figure 6E) and did not gain AM-like affinity for the AM_1 receptor ECD complex (Figure 6H). CGRPmut [F37Y] retained CGRP receptor ECD complex binding (Figure 6F) and gained the ability to bind the AM_1 receptor ECD complex as strongly as AM (Figure 6I). AM(37-52)NH_2 [Y52F] exhibited significantly diminished binding to the AM_1 receptor ECD complex (Figure 6J) but did not gain increased affinity for the CGRP receptor ECD complex (Figure 6G). These results suggested that the RAMP2 E101-AM Y52 hydrogen bond is a key contributor to AM_1 receptor selectivity, whereas Phe as the peptide C-terminal residue is insufficient to confer CGRP receptor selectivity.

DISCUSSION

RAMPs are an important class of accessory membrane proteins that modulate GPCR pharmacology. The CGRPmut-bound CLR:RAMP1 ECD and AM-bound CLR:RAMP2 ECD structures presented here expand our understanding of the mechanisms by which peptides can bind to class B GPCRs and increase our understanding of how RAMPs enable peptide selectivity. The engineered tethered ECD fusion proteins used for crystallization exhibited the same peptide selectivity rank order as the intact receptors, which indicated that they are valid reagents for studying selective peptide binding. The purified proteins bound their respective peptides with apparent affinities in the low μM range (Figures S1C, S1G, and S1H), which are lower than the affinities of the agonist peptides for intact receptors but typical for truncated peptides at class B GPCR ECDs (Pal et al., 2010; Parthier et al., 2007; Pioszak and Xu, 2008).

The oligomeric states of CLR:RAMP complexes has been a source of debate with evidence for 1:1, 2:1, and 2:2 CLR:RAMP stoichiometries (Héroux et al., 2007; Hill and Pioszak, 2013;

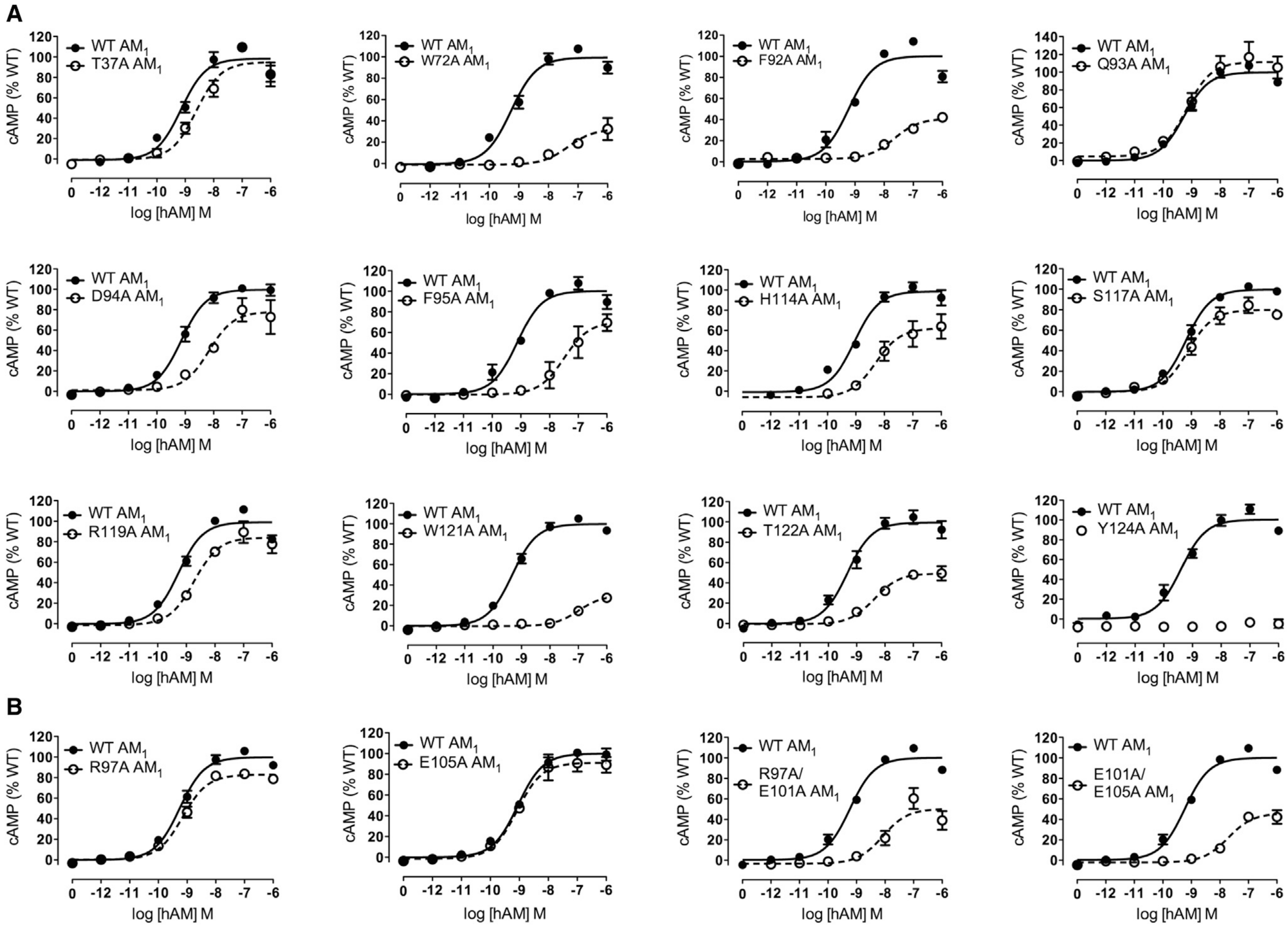

Figure 5. Validation of the AM-Bound ECD Heterodimer Structure for the Intact AM$_1$ Receptor Transiently Expressed in COS-7 Cells
Concentration-response curves for each of the CLR (A) or RAMP2 (B) alanine substitution mutants tested with hAM in cAMP signaling assays. Data are represented as mean ± SEM. See also Table S4.

Kusano et al., 2012; Moad and Pioszak, 2013; Watkins et al., 2013a). Dimerization of the purified CLR:RAMP2 ECD heterodimer to form a 2:2 complex may be an artifact because the RAMP2 L106R mutation prevented oligomerization yet did not affect AM$_1$ receptor function (Figure S1). Occlusion of the AM-binding site by dimerization explains our inability to measure AM binding to the tethered RAMP2-CLR ECD fusion protein in an assay using μM receptor concentration, whereas AM binding was readily measured in an assay using nM receptor concentration where the dimeric species was likely not significantly present (Moad and Pioszak, 2013). Héroux et al. (2007) provided evidence for a homo-oligomer of CLR with a single RAMP1 as the functional CGRP receptor in cells. We cannot rule out a role for higher-order oligomerization in the function of the intact receptors, but the structures indicate that the 1:1 heterodimers are sufficient to bind peptides.

The CGRPmut and AM peptides adopted receptor-bound conformations different from typical α-helical class B GPCR peptide ligands. CGRPmut and AM are characterized by a shared turn structure that positions their C-terminal residue to occupy the pocket near the RAMP. Previous studies indicated the presence of turns in the C-terminal region of CGRP (and an absence of α-helix in this area), but how this region interacted with the receptor was unclear (Carpenter et al., 2001; Watkins et al., 2013b). Prior to the turns, the peptides diverge in their structure and interactions with CLR, but they contact the same area of CLR with little or no peptide secondary structure. NMR structures of CGRP and AM suggested that α-helix is restricted to residues 8-18/22-34 of these peptides (Boulanger et al., 1995; Breeze et al., 1991; Pérez-Castells et al., 2012; Watkins et al., 2013a) and indeed the C-terminal regions of both contain helix-breaking Pro residues (Figure 1D). Accordingly, the lack of substantial helical content in the bound peptide fragments is consistent with what is known about the structures of the full-length peptides. A turn structure and paucity of α-helicity may be a general feature of the receptor ECD-binding portions of CT family peptides.

The CGRPmut and AM binding modes are consistent with peptide mutagenesis studies. CGRP T30, V32, and F37 and the C-terminal amide were important for binding purified

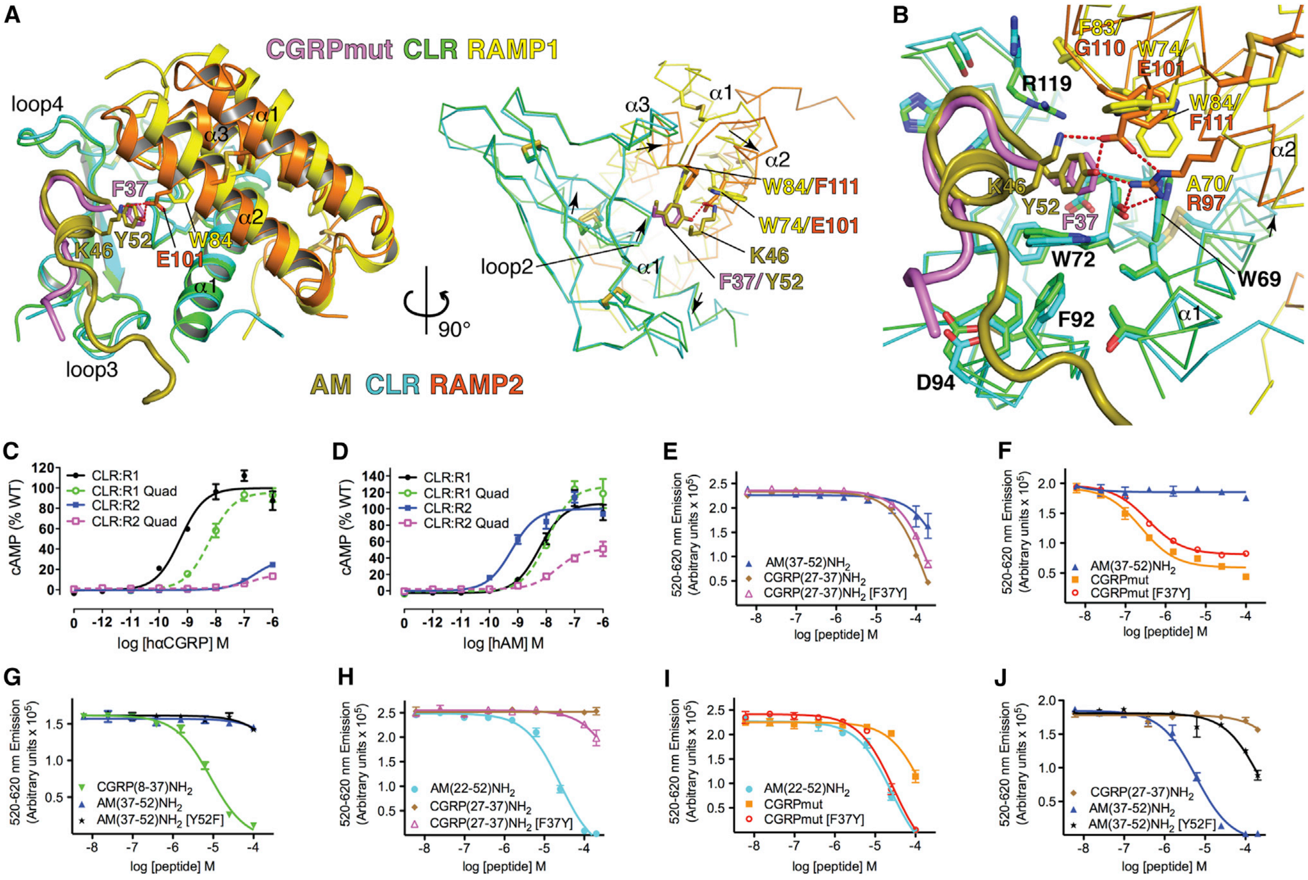

Figure 6. Peptide Selectivity Determinants for CLR:RAMP1/2 Complexes

(A) Superposition of the CGRPmut- and AM-bound ECD heterodimers aligned based on the CLR positions. The receptors and peptides are in cartoon representation with selected residues as sticks in the left image. In the right image, the receptors are Cα traces and the peptide cartoons were omitted for clarity. Arrows indicate directions of movement of CLR α1 and loop 2 and RAMP α2 and α3 from the CGRPmut/RAMP1-bound state to the AM/RAMP2-bound state. Red dashes are hydrogen bonds.

(B) Detailed view of the aligned CGRPmut- and AM-bound complexes with selected residues as sticks and the receptors as Cα traces. The arrow highlights the shift of the C-terminal region of CLR α1 from the CGRPmut/RAMP1- to AM/RAMP2-bound states.

(C and D) Concentration-response curves for the CGRP and AM_1 receptors with the RAMP1 (A70R, W74E, F83G, W84F) and RAMP2 (R97A, E101W, G110F, F111W) quadruple "swap" mutants tested with hαCGRP and hAM in cAMP assays in COS-7 cells. The cell surface expression was: RAMP1 quad 116.4 ± 5.25 (n = 4) % WT, RAMP2 quad 73.7 ± 9.52 (n = 4) % WT p < 0.01 by one-way ANOVA followed by Dunnett's test for the RAMP2 quad mutant.

(E–J) Competition AlphaScreen assays with purified receptor ECD heterodimer proteins and the indicated competitor "swap" peptides. (E)–(G) are for the MBP-RAMP1-$(GSA)_3$-CLR-H_6 protein with biotin-CGRP (100 nM each) and (H)–(J) are for the MBP-RAMP2[L106R]-$(GSA)_3$-CLR-H_6 protein with biotin-AM (100 nM each). The binding data are representative of at least three independent experiments each performed in duplicate. The error bars represent the SEM of the experiment. Determinable pIC_{50} values were as follows: (F), CGRPmut 6.79 ± 0.10 and CGRPmut [F37Y] 6.24 ± 0.09; (G), CGRP(8-37) 5.01 ± 0.04; (H), AM(22-52) 4.92 ± 0.16; (I), AM(22-52) 4.84 ± 0.09 and CGRPmut [F37Y] 4.79 ± 0.07; (J), AM(37-52) 5.00 ± 0.11.

CLR:RAMP1 ECD (Moad and Pioszak, 2013) and intact CGRP receptor (Carpenter et al., 2001; Rist et al., 1998; Watkins et al., 2013b). Modified CGRP peptides as short as 30–37 maintained the ability to bind the receptor (Carpenter et al., 2001), consistent with this region providing most of the contacts. Increased affinity of CGRPmut over that of CGRP can be explained by their differences in the turn region (Figure 1D). P34 favors β-turn formation better than S34 and F35 provides better hydrophobic contact to CLR loop 4 than K35. AM P43, K46, I47, G51, Y52, and the C-terminal amide were most critical for binding purified CLR:RAMP2 ECD and intact AM_1 receptor, and truncation beyond residue 38 diminished binding even though the K38-V41 side chains were not important (Moad and Pioszak, 2013; Watkins et al., 2013a).

Mapping the receptor mutagenesis data (Figures 4 and 5) onto the surface of the receptor structures (Figures 7A and 7B) suggests that CGRP binds in a similar manner to CGRPmut and that the structures are good models for full-length CGRP and AM binding to intact receptors. Mutation of CLR residues that form the shared binding site diminished CGRP and AM potencies, and the effects of some of the mutations were similar for both peptides (e.g., F92). Noteworthy divergent effects of several mutations support the differences in the structures. CLR D94 was far more important for CGRP action than AM,

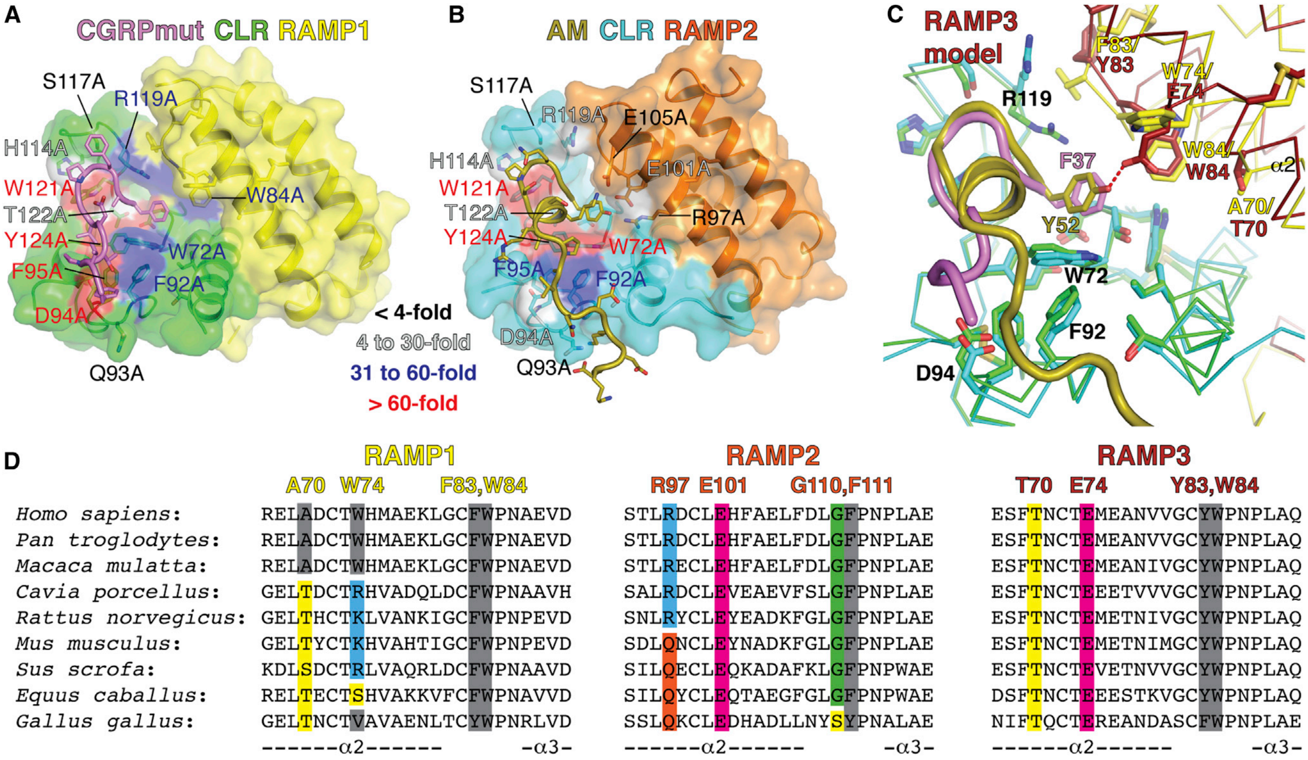

Figure 7. Summary of Peptide Recognition and Selectivity Determinants for CLR:RAMP1-3 Complexes

(A and B) Structures of the CGRPmut- and AM-bound CLR:RAMP1/2 ECD heterodimers with the surface of receptor residues colored according to their effect on CGRP (A) or AM (B) signaling potency when mutated to alanine. Color coding signifies the extent of reduced signaling potency at intact receptor complexes in cells compared to wild-type as indicated by the inset legend. RAMP1 W84A data are from Moore et al. (2010) (34-fold reduced potency).

(C) Model for RAMP3 binding site augmentation. A homology model of the AM-bound CLR:RAMP3 ECD complex (Supplemental Experimental Procedures) was superimposed with the CGRPmut-bound CLR:RAMP1 and AM-bound CLR:RAMP2 ECD structures based on the CLR positions. Only the RAMP3 subunit from the homology model is shown and RAMP2 is omitted. The receptors are shown as Cα traces and selected residues as sticks.

(D) Amino acid sequence alignments for RAMP1-3 from the indicated species showing the α2 and α2-α3 loop regions that augment the peptide-binding site.

consistent with the crucial role of D94 in contacting CGRP T30 and its less important role in contacting the AM main chain. CLR R119A diminished the potency of CGRP much more than that of AM, which may reflect an important role for the different R119 conformations observed in the two structures. CLR W72A was more deleterious for AM action than CGRP, which is consistent with the greater number of AM contacts to CLR W72 as compared to CGRP.

RAMP1 W84 and RAMP2 E101 were previously identified as key residues for CGRP and AM function, respectively (Moore et al., 2010; Watkins et al., 2014). These data are explained by how these residues augment the binding site pocket (Figures 7A and 7B). Apparently, packing of the CGRP F37 and AM Y52 phenyl rings against the CLR Trp shelf and G71 is insufficient for strong binding. RAMP1 W84 or RAMP2 E101 is required to complete the pocket to enable strong "anchoring" of the peptide C termini. RAMP2 R97 and E105 also augment the pocket, but the R97A and E105A mutants did not diminish AM potency. These data along with the peptide swap data indicate that the RAMP2 E101-Y52 hydrogen bond is the crucial AM anchoring contact. Ionic interactions of AM K46 and RAMP2 E101/E105 do not appear to be significant. The main role of AM K46 thus appears to be intramolecular packing against Y52 and contacting the Trp shelf.

Distinct RAMP binding site augmentation clearly contributes to peptide selectivity (Figure 6). RAMP2 E101 favors AM binding because it can hydrogen bond with Y52 and RAMP2 F111 discourages CGRP binding because it is too small to contact the F37 phenyl ring. Indeed, the F37Y swap in CGRPmut conferred strong affinity for the AM_1 receptor ECD complex and the Y52F swap in AM(37-52)NH_2 significantly diminished its binding. The lack of Glu at RAMP1 position 74 would disfavor strong AM binding. RAMP1 W84 enables strong CGRP binding by contacting F37, but this contact alone is apparently insufficient for selectivity because AM(37-52)NH_2 [Y52F] did not gain affinity for the CGRP receptor ECD complex.

Modeling the AM-bound AM_2 receptor ECD complex (Supplemental Experimental Procedures) suggests that RAMP3 augments the binding site as a RAMP1-2 hybrid (Figure 7C). RAMP3 E74, which is equivalent to RAMP2 E101, would favor AM binding by hydrogen bonding with Y52. RAMP3 W84, which is equivalent to RAMP1 W84, could contact the AM Y52 and CGRP F37 phenyl rings, thereby explaining diminished potency of both peptides at the AM_2 receptor with RAMP3 W84A and why

CGRP is more active at the AM_2 receptor than the AM_1 receptor (Watkins et al., 2014). The key RAMP residues proposed as selectivity determinants are conserved across species: W84 and a lack of Glu at position 74 in RAMP1, E101 and F or Y at position 111 in RAMP2, and E74 and W84 in RAMP3 (Figure 7D). A small amino acid is conserved at position 70 in RAMP1/3, which would avoid steric clash with W84. Notably, the lack of conservation of RAMP2 R97 and E105 is consistent with the mutagenesis data that indicated that these residues are not critical for AM signaling.

Previous RAMP single swap mutant studies supported the importance of Glu at position 74/101 as a determinant for AM selectivity. RAMP1 W74E had no effect on CGRP potency but increased AM potency at the CGRP receptor (Qi et al., 2008, 2011). RAMP3 E74W decreased AM potency at the AM_2 receptor, while having a negligible effect on CGRP potency (Hay et al., 2006; Qi et al., 2008, 2011). More extensive quadruple swap mutants in this study failed to exchange the pharmacological profiles, but these experiments are complicated by the different RAMP positions relative to CLR and variable RAMP effects on CLR conformation.

The failure of the CGRP and AM peptide C-terminal residue swaps to exchange their receptor preferences (Figure 6) strongly suggests that RAMP binding site augmentation alone is insufficient to account for selectivity. Thus, the subtle differences in CLR conformation in the two structures may also be important for selectivity. RAMP-induced changes in CLR R119 side-chain conformation and/or subtle shifting of loop 2 may sufficiently alter the pocket to favor one peptide over the other. Future studies will be required to determine to what extent such allostery contributes to selectivity. Peptide selectivity determinants may also exist in portions of the receptors that were not addressed in this study.

In summary, the structures presented here provide the first structural views of any accessory membrane protein modulating GPCR ligand binding and may provide a basis for understanding modulation of other GPCRs by accessory proteins. Our data indicate that RAMPs determine peptide selectivity of CLR through a combination of binding site augmentation and alteration of CLR conformation. It is striking that relatively minor differences in RAMP-specific peptide contacts and subtle RAMP-induced changes in CLR conformation lead to such profoundly different pharmacological profiles. Of practical value, the structures may inform rational drug design targeting CLR:RAMP complexes with clinical relevance for migraine headache and cardiovascular disorders. Lastly, the MBP-tethered ECD fusion protein approach to crystallization should facilitate structural studies of other CT family peptides bound to their respective receptor ECD complexes, which will enable a more complete understanding of how RAMPs modulate both CLR and CTR.

EXPERIMENTAL PROCEDURES

Protein Production and Characterization and Peptides

Plasmid construction, mutagenesis, protein expression, purification, and the AlphaScreen peptide-binding assay were as previously described with minor modifications to the AlphaScreen assay (Hill and Pioszak, 2013; Moad and Pioszak, 2013). Synthetic peptides were from RS Synthesis, Bachem, or were synthesized in-house. Details are in Supplemental Experimental Procedures.

Crystallization, Structure Solution, and Homology Modeling

The tethered MBP-RAMP1 ECD-CLR ECD and MBP-RAMP2 ECD [L106R]-CLR ECD proteins were complexed with CGRP(27-37)NH_2 [D31, P34, F35] or AM(25-52)NH_2 and crystallized with a reservoir solution of 22% PEG3350, 8% Tacsimate (pH 6.0) for the CGRP receptor complex or 19% PEG3350, 0.1 M Tris-HCl (pH 8.3), 225 mM sodium acetate, and 20% ethylene glycol for the AM_1 receptor complex. Diffraction data collected at the APS synchrotron were processed with HKL2000 (Otwinowski and Minor, 1997) and the CCP4 suite (Winn et al., 2011). The structures were solved by molecular replacement with Phaser (McCoy et al., 2007), rebuilt with COOT (Emsley et al., 2010), and refined with REFMAC5 (Murshudov et al., 1997). Details and homology modeling are in Supplemental Experimental Procedures.

Cell-Based Assays

Transfection of COS-7 cells, cAMP assay, ELISA for cell surface expression, and data analysis were as previously described (Barwell et al., 2010; Watkins et al., 2014).

ACCESSION NUMBERS

The accession numbers for the coordinates and structure factors reported in this paper for the AM:CLR:RAMP2 and CGRPmut:CLR:RAMP1 complexes, respectively are PDB: 4RWF, 4RWG.

SUPPLEMENTAL INFORMATION

Supplemental Information includes Supplemental Experimental Procedures, two figures, and four tables and can be found with this article online at http://dx.doi.org/10.1016/j.molcel.2015.04.018.

AUTHOR CONTRIBUTIONS

J.M.B. produced and characterized proteins, performed crystallization, collected diffraction data, and constructed CLR and RAMP mutants; C.S.W., M.A.J., and D.L.H. designed, performed, and analyzed cell-based assays for the CGRP and AM_1 receptors; J.B., G.K., J.S., R.M.B., and D.R.P. designed and constructed CLR mutants and performed and analyzed cell-based assays for the CGRP receptor; J.S. performed RAMP3 homology modeling; M.L.W. performed peptide binding experiments; P.W.H. and M.A.B. synthesized CGRP analog; A.A.P. designed and managed the structural project, solved/refined the structures, and wrote the manuscript with D.R.P. and D.L.H.

ACKNOWLEDGMENTS

This research was supported by NIH grant R01GM104251 (A.A.P.), Wellcome Trust grant 091496 (D.R.P.), and grants from the Marsden Fund and Health Research Council of New Zealand (D.L.H.). Use of the Leica microscope for crystal imaging was supported by an Institutional Developmental Award from the National Institute of General Medical Sciences of the NIH under grant number P20GM103640. Use of the Advanced Photon Source, an Office of Science User Facility operated for the U.S. Department of Energy (DOE) Office of Science by Argonne National Laboratory, was supported by the U.S. DOE under Contract No. DE-AC02-06CH11357. Use of the LS-CAT Sector 21 was supported by the Michigan Economic Development Corporation and the Michigan Technology Tri-Corridor (Grant 085P1000817). We thank Zdzislaw Wawrzak and Spencer Anderson for assistance with remote data collection at APS sector 21 and Amanda Roehrkasse for pilot studies with the tethered RAMP1-$(GSA)_3$-CLR construct.

Received: December 23, 2014
Revised: March 23, 2015
Accepted: April 9, 2015
Published: May 14, 2015

REFERENCES

Archbold, J.K., Flanagan, J.U., Watkins, H.A., Gingell, J.J., and Hay, D.L. (2011). Structural insights into RAMP modification of secretin family G protein-coupled receptors: implications for drug development. Trends Pharmacol. Sci. *32*, 591–600.

Barwell, J., Miller, P.S., Donnelly, D., and Poyner, D.R. (2010). Mapping interaction sites within the N-terminus of the calcitonin gene-related peptide receptor; the role of residues 23-60 of the calcitonin receptor-like receptor. Peptides *31*, 170–176.

Boulanger, Y., Khiat, A., Chen, Y., Senécal, L., Tu, Y., St-Pierre, S., and Fournier, A. (1995). Structure of human calcitonin gene-related peptide (hCGRP) and of its antagonist hCGRP 8-37 as determined by NMR and molecular modeling. Pept. Res. *8*, 206–213.

Bouschet, T., Martin, S., and Henley, J.M. (2005). Receptor-activity-modifying proteins are required for forward trafficking of the calcium-sensing receptor to the plasma membrane. J. Cell Sci. *118*, 4709–4720.

Breeze, A.L., Harvey, T.S., Bazzo, R., and Campbell, I.D. (1991). Solution structure of human calcitonin gene-related peptide by 1H NMR and distance geometry with restrained molecular dynamics. Biochemistry *30*, 575–582.

Carpenter, K.A., Schmidt, R., von Mentzer, B., Haglund, U., Roberts, E., and Walpole, C. (2001). Turn structures in CGRP C-terminal analogues promote stable arrangements of key residue side chains. Biochemistry *40*, 8317–8325.

Christopoulos, G., Perry, K.J., Morfis, M., Tilakaratne, N., Gao, Y., Fraser, N.J., Main, M.J., Foord, S.M., and Sexton, P.M. (1999). Multiple amylin receptors arise from receptor activity-modifying protein interaction with the calcitonin receptor gene product. Mol. Pharmacol. *56*, 235–242.

Durham, P.L., and Vause, C.V. (2010). Calcitonin gene-related peptide (CGRP) receptor antagonists in the treatment of migraine. CNS Drugs *24*, 539–548.

Edelman, S., Maier, H., and Wilhelm, K. (2008). Pramlintide in the treatment of diabetes mellitus. BioDrugs *22*, 375–386.

Emsley, P., Lohkamp, B., Scott, W.G., and Cowtan, K. (2010). Features and development of Coot. Acta Crystallogr. D Biol. Crystallogr. *66*, 486–501.

Hay, D.L., Christopoulos, G., Christopoulos, A., and Sexton, P.M. (2006). Determinants of 1-piperidinecarboxamide, N-[2-[[5-amino-l-[[4-(4-pyridinyl)-l-piperazinyl]carbonyl]pentyl]amino]-1-[(3,5-dibromo-4-hydroxyphenyl) methyl]-2-oxoethyl]-4-(1,4-dihydro-2-oxo-3(2H)-quinazolinyl) (BIBN4096BS) affinity for calcitonin gene-related peptide and amylin receptors—the role of receptor activity modifying protein 1. Mol. Pharmacol. *70*, 1984–1991.

Héroux, M., Hogue, M., Lemieux, S., and Bouvier, M. (2007). Functional calcitonin gene-related peptide receptors are formed by the asymmetric assembly of a calcitonin receptor-like receptor homo-oligomer and a monomer of receptor activity-modifying protein-1. J. Biol. Chem. *282*, 31610–31620.

Hill, H.E., and Pioszak, A.A. (2013). Bacterial expression and purification of a heterodimeric adrenomedullin receptor extracellular domain complex using DsbC-assisted disulfide shuffling. Protein Expr. Purif. *88*, 107–113.

Hoare, S.R. (2005). Mechanisms of peptide and nonpeptide ligand binding to Class B G-protein-coupled receptors. Drug Discov. Today *10*, 417–427.

Hong, Y., Hay, D.L., Quirion, R., and Poyner, D.R. (2012). The pharmacology of adrenomedullin 2/intermedin. Br. J. Pharmacol. *166*, 110–120.

Karpinich, N.O., Hoopes, S.L., Kechele, D.O., Lenhart, P.M., and Caron, K.M. (2011). Adrenomedullin function in vascular endothelial cells: insights from genetic mouse models. Curr. Hypertens. Rep. *7*, 228–239.

Kumar, S., Pioszak, A., Zhang, C., Swaminathan, K., and Xu, H.E. (2011). Crystal structure of the PAC1R extracellular domain unifies a consensus fold for hormone recognition by class B G-protein coupled receptors. PLoS ONE *6*, e19682.

Kusano, S., Kukimoto-Niino, M., Hino, N., Ohsawa, N., Okuda, K., Sakamoto, K., Shirouzu, M., Shindo, T., and Yokoyama, S. (2012). Structural basis for extracellular interactions between calcitonin receptor-like receptor and receptor activity-modifying protein 2 for adrenomedullin-specific binding. Protein Sci. *21*, 199–210.

Lenhart, P.M., Broselid, S., Barrick, C.J., Leeb-Lundberg, L.M., and Caron, K.M. (2013). G-protein-coupled receptor 30 interacts with receptor activity-modifying protein 3 and confers sex-dependent cardioprotection. J. Mol. Endocrinol. *51*, 191–202.

McCoy, A.J., Grosse-Kunstleve, R.W., Adams, P.D., Winn, M.D., Storoni, L.C., and Read, R.J. (2007). Phaser crystallographic software. J. Appl. Cryst. *40*, 658–674.

McLatchie, L.M., Fraser, N.J., Main, M.J., Wise, A., Brown, J., Thompson, N., Solari, R., Lee, M.G., and Foord, S.M. (1998). RAMPs regulate the transport and ligand specificity of the calcitonin-receptor-like receptor. Nature *393*, 333–339.

Moad, H.E., and Pioszak, A.A. (2013). Selective CGRP and adrenomedullin peptide binding by tethered RAMP-calcitonin receptor-like receptor extracellular domain fusion proteins. Protein Sci. *22*, 1775–1785.

Moore, E.L., Gingell, J.J., Kane, S.A., Hay, D.L., and Salvatore, C.A. (2010). Mapping the CGRP receptor ligand binding domain: tryptophan-84 of RAMP1 is critical for agonist and antagonist binding. Biochem. Biophys. Res. Commun. *394*, 141–145.

Murshudov, G.N., Vagin, A.A., and Dodson, E.J. (1997). Refinement of macromolecular structures by the maximum-likelihood method. Acta Crystallogr. D Biol. Crystallogr. *53*, 240–255.

Otwinowski, Z., and Minor, W. (1997). Processing of X-ray diffraction data collected in oscillation mode. In Methods in Enzymology, C.W.J. Carter and R.M. Sweet, eds. (New York: Academic Press), pp. 307–326.

Pal, K., Swaminathan, K., Xu, H.E., and Pioszak, A.A. (2010). Structural basis for hormone recognition by the Human CRFR2alpha G protein-coupled receptor. J. Biol. Chem. *285*, 40351–40361.

Parameswaran, N., and Spielman, W.S. (2006). RAMPs: The past, present and future. Trends Biochem. Sci. *31*, 631–638.

Parthier, C., Kleinschmidt, M., Neumann, P., Rudolph, R., Manhart, S., Schlenzig, D., Fanghänel, J., Rahfeld, J.U., Demuth, H.U., and Stubbs, M.T. (2007). Crystal structure of the incretin-bound extracellular domain of a G protein-coupled receptor. Proc. Natl. Acad. Sci. USA *104*, 13942–13947.

Pérez-Castells, J., Martín-Santamaría, S., Nieto, L., Ramos, A., Martínez, A., Pascual-Teresa, Bd., and Jiménez-Barbero, J. (2012). Structure of micelle-bound adrenomedullin: a first step toward the analysis of its interactions with receptors and small molecules. Biopolymers *97*, 45–53.

Pioszak, A.A., and Xu, H.E. (2008). Molecular recognition of parathyroid hormone by its G protein-coupled receptor. Proc. Natl. Acad. Sci. USA *105*, 5034–5039.

Pioszak, A.A., Parker, N.R., Suino-Powell, K., and Xu, H.E. (2008). Molecular recognition of corticotropin-releasing factor by its G-protein-coupled receptor CRFR1. J. Biol. Chem. *283*, 32900–32912.

Pioszak, A.A., Parker, N.R., Gardella, T.J., and Xu, H.E. (2009). Structural basis for parathyroid hormone-related protein binding to the parathyroid hormone receptor and design of conformation-selective peptides. J. Biol. Chem. *284*, 28382–28391.

Pioszak, A.A., Harikumar, K.G., Parker, N.R., Miller, L.J., and Xu, H.E. (2010). Dimeric arrangement of the parathyroid hormone receptor and a structural mechanism for ligand-induced dissociation. J. Biol. Chem. *285*, 12435–12444.

Poyner, D.R., Sexton, P.M., Marshall, I., Smith, D.M., Quirion, R., Born, W., Muff, R., Fischer, J.A., and Foord, S.M. (2002). International Union of Pharmacology. XXXII. The mammalian calcitonin gene-related peptides, adrenomedullin, amylin, and calcitonin receptors. Pharmacol. Rev. *54*, 233–246.

Purdue, B.W., Tilakaratne, N., and Sexton, P.M. (2002). Molecular pharmacology of the calcitonin receptor. Receptors Channels *8*, 243–255.

Qi, T., and Hay, D.L. (2010). Structure-function relationships of the N-terminus of receptor activity-modifying proteins. Br. J. Pharmacol. *159*, 1059–1068.

Qi, T., Christopoulos, G., Bailey, R.J., Christopoulos, A., Sexton, P.M., and Hay, D.L. (2008). Identification of N-terminal receptor activity-modifying protein residues important for calcitonin gene-related peptide, adrenomedullin, and amylin receptor function. Mol. Pharmacol. *74*, 1059–1071.

Qi, T., Ly, K., Poyner, D.R., Christopoulos, G., Sexton, P.M., and Hay, D.L. (2011). Structure-function analysis of amino acid 74 of human RAMP1 and RAMP3 and its role in peptide interactions with adrenomedullin and calcitonin gene-related peptide receptors. Peptides *32*, 1060–1067.

Rist, B., Entzeroth, M., and Beck-Sickinger, A.G. (1998). From micromolar to nanomolar affinity: a systematic approach to identify the binding site of CGRP at the human calcitonin gene-related peptide 1 receptor. J. Med. Chem. *41*, 117–123.

Runge, S., Thøgersen, H., Madsen, K., Lau, J., and Rudolph, R. (2008). Crystal structure of the ligand-bound glucagon-like peptide-1 receptor extracellular domain. J. Biol. Chem. *283*, 11340–11347.

ter Haar, E., Koth, C.M., Abdul-Manan, N., Swenson, L., Coll, J.T., Lippke, J.A., Lepre, C.A., Garcia-Guzman, M., and Moore, J.M. (2010). Crystal structure of the ectodomain complex of the CGRP receptor, a class-B GPCR, reveals the site of drug antagonism. Structure *18*, 1083–1093.

Underwood, C.R., Garibay, P., Knudsen, L.B., Hastrup, S., Peters, G.H., Rudolph, R., and Reedtz-Runge, S. (2010). Crystal structure of glucagon-like peptide-1 in complex with the extracellular domain of the glucagon-like peptide-1 receptor. J. Biol. Chem. *285*, 723–730.

Watkins, H.A., Au, M., Bobby, R., Archbold, J.K., Abdul-Manan, N., Moore, J.M., Middleditch, M.J., Williams, G.M., Brimble, M.A., Dingley, A.J., and Hay, D.L. (2013a). Identification of key residues involved in adrenomedullin binding to the AM1 receptor. Br. J. Pharmacol. *169*, 143–155.

Watkins, H.A., Rathbone, D.L., Barwell, J., Hay, D.L., and Poyner, D.R. (2013b). Structure-activity relationships for α-calcitonin gene-related peptide. Br. J. Pharmacol. *170*, 1308–1322.

Watkins, H.A., Walker, C.S., Ly, K.N., Bailey, R.J., Barwell, J., Poyner, D.R., and Hay, D.L. (2014). Receptor activity-modifying protein-dependent effects of mutations in the calcitonin receptor-like receptor: implications for adrenomedullin and calcitonin gene-related peptide pharmacology. Br. J. Pharmacol. *171*, 772–788.

Winn, M.D., Ballard, C.C., Cowtan, K.D., Dodson, E.J., Emsley, P., Evans, P.R., Keegan, R.M., Krissinel, E.B., Leslie, A.G., McCoy, A., et al. (2011). Overview of the CCP4 suite and current developments. Acta Crystallogr. D Biol. Crystallogr. *67*, 235–242.

Wootten, D.L., Simms, J., Hay, D.L., Christopoulos, A., and Sexton, P.M. (2010). Receptor activity modifying proteins and their potential as drug targets. Prog. Mol. Biol. Transl. Sci. *91*, 53–79.

Molecular Cell

Supplemental Information

Structural Basis for Receptor Activity-Modifying Protein-Dependent Selective Peptide Recognition by a G Protein-Coupled Receptor

Jason M. Booe, Christopher Walker, James Barwell, Gabriel Kuteyi, John Simms, Muhammad A. Jamaluddin, Margaret L. Warner, Roslyn M. Bill, Paul W. Harris, Margaret A. Brimble, David R. Poyner, Debbie L. Hay, and Augen A. Pioszak

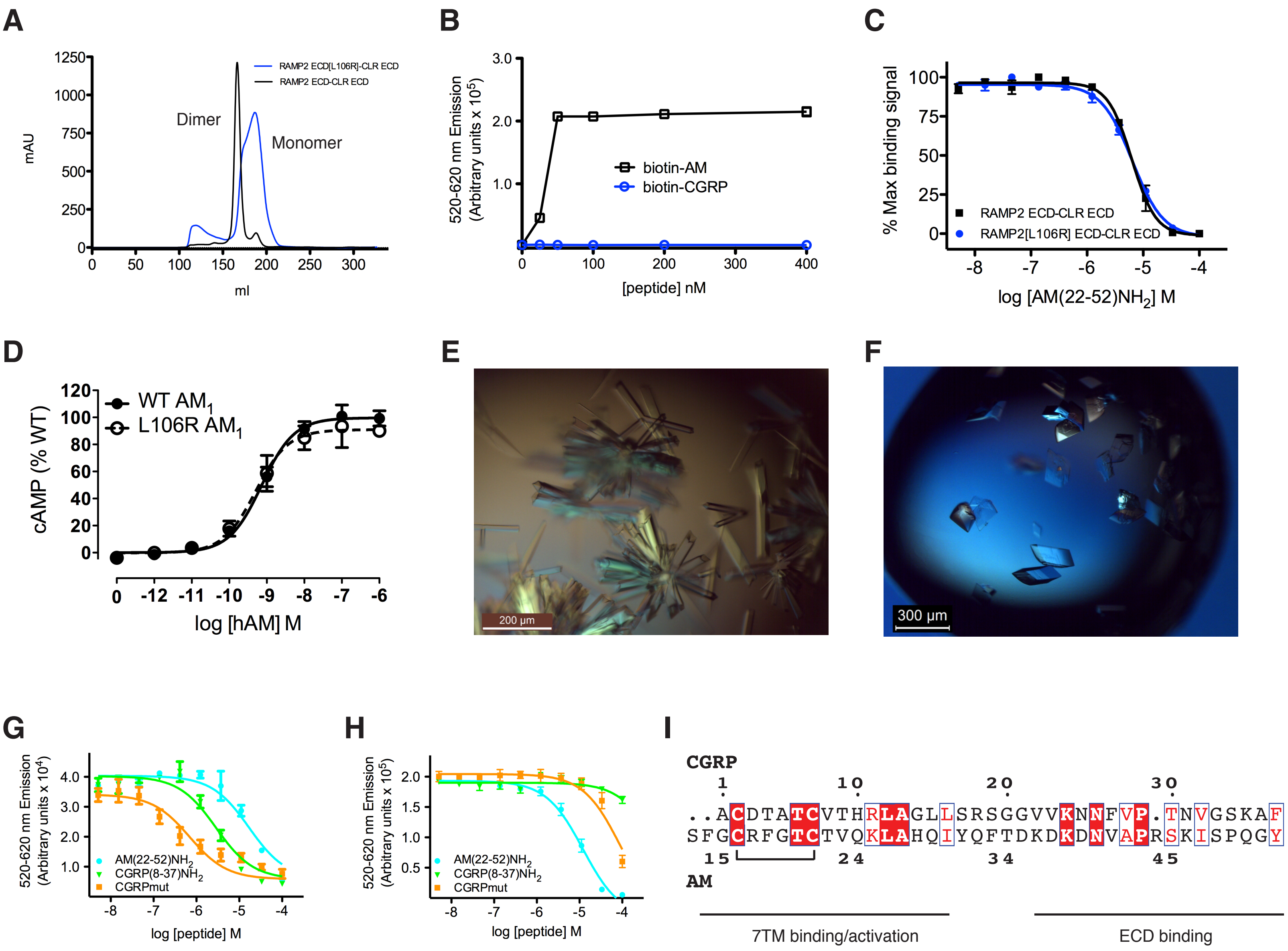

Supplemental Figure S1 (related to Figure 1). Protein characterization and crystallization. (A) Superdex200 HR gel-filtration elution profiles for MBP-RAMP2 ECD-$(GS)_5$-CLR ECD-H_6 and MBP-RAMP2 ECD [L106R]-$(GSA)_3$-CLR ECD-H_6 tethered fusion proteins. (B) AlphaScreen assay in saturation binding format for the RAMP2 [L106R] tethered fusion (150 nM) with the indicated concentrations of N-terminally biotinylated AM or CGRP. (C) Competition AlphaScreen assay for the indicated tethered fusion proteins (125 nM) and biotin-AM (125 nM) incubated with the indicated concentrations of AM(22-52)NH_2 competitor peptide. Data shown are representative of three independent experiments each performed in duplicate. The pIC_{50} values for the WT RAMP2 ECD-CLR ECD fusion and the RAMP2 [L106R]-CLR ECD fusion were 5.32 ± 0.08 and 5.27 ± 0.09, respectively. (D) Concentration-response curve for AM_1 receptor with the RAMP2 [L106R] mutant tested with hAM in a cAMP assay in COS-7 cells. (E) Crystals of the AM(25-52)NH_2-bound MBP-RAMP2 ECD [L106R]-$(GSA)_3$-CLR ECD-H_6 fusion. Individual rods were broken off the clusters for data collection. (F) Crystals of the CGRPmut-bound MBP-RAMP1 ECD-$(GSA)_3$-CLR ECD-H_6 fusion protein. (G) Competition AlphaScreen peptide binding assay for the MBP-RAMP1 ECD-$(GSA)_3$-CLR ECD-H_6 fusion protein (100 nM) and biotin-CGRP (100 nM) incubated with the indicated competitor peptides. The pIC_{50} values for AM(22-52)NH_2, CGRP(8-37)NH_2, and CGRPmut were 4.99 ± 0.13, 5.67 ± 0.10, 6.34 ± 0.09, respectively. (H) Competition AlphaScreen assay for the MBP-RAMP2 ECD [L106R]-$(GSA)_3$-CLR ECD-H_6 fusion protein (100 nM) and biotin-AM (100 nM) incubated with the indicated competitor peptides. The pIC_{50} value for AM(22-52)NH_2 was 4.89 ± 0.01. (I) Amino acid sequence alignment of human αCGRP and AM.

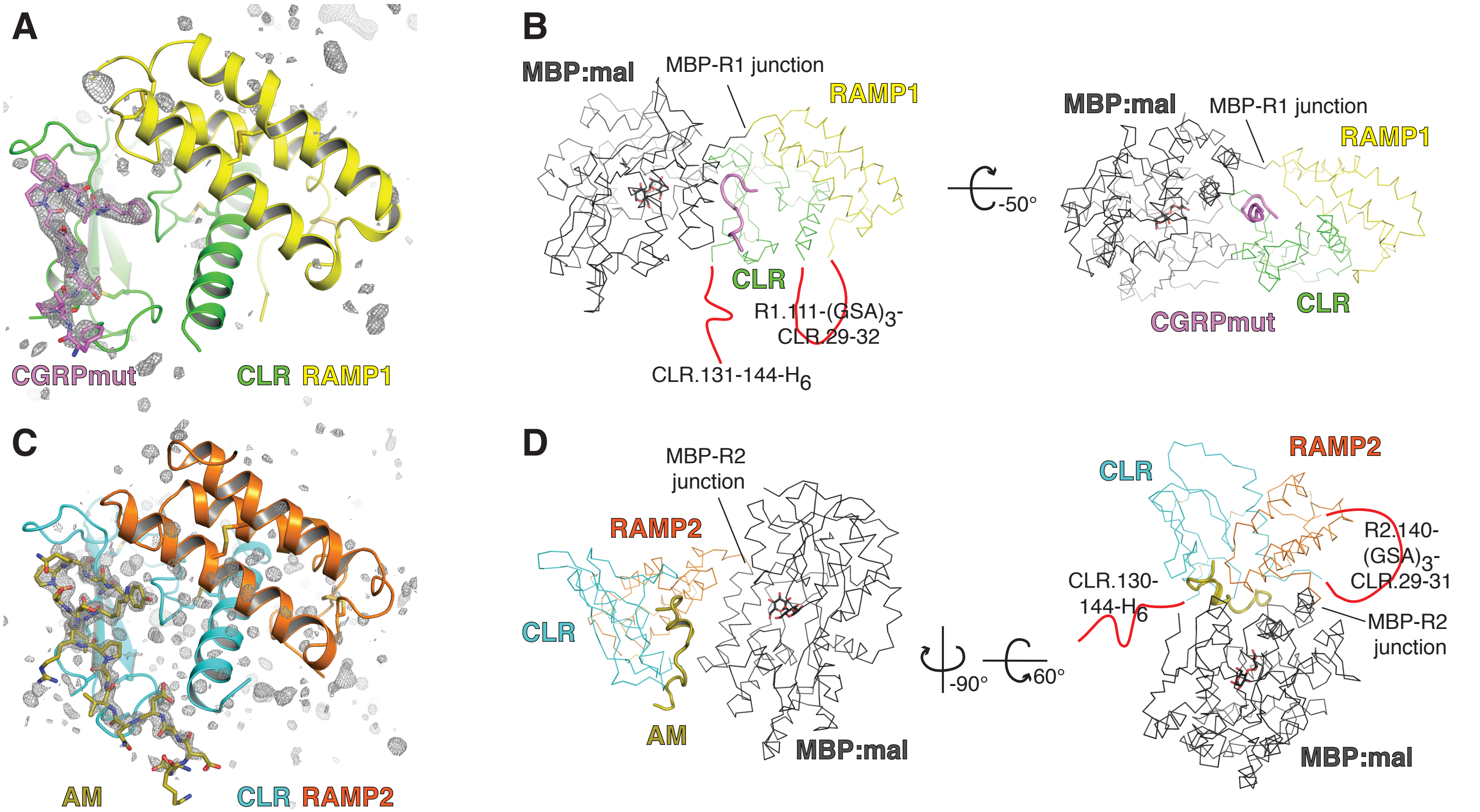

Supplemental Figure S2 (related to Figure 1). Electron density maps and structures of the MBP-tethered ECD fusion proteins. (A, C) The CGRP and AM_1 receptor ECD complex structures obtained after initial rebuilding of the molecular replacement models, but before addition of peptides or water molecules are shown with the mF_o-DF_c electron density maps (gray mesh) from these models contoured at 3σ. The CGRPmut and AM peptides from the final refined structures are included for reference. (B, D) Structures of the tethered fusion proteins highlighting the positions of MBP relative to the ECD complexes and disordered tethers and C-terminal regions (red lines). Molecule A is shown for the CGRP receptor structure. In the left image of panel B the CGRP receptor ECD complex is oriented the same as in panel A. In the left image of panel D the AM_1 receptor ECD complex is oriented the same as in Figure 1B, right image.

Table S1 (related to Fig 2). Summary of contacts between CGRP [D31, P34, F35] and CLR:RAMP1 ECD.

CGRP residue	Contact with CLR	Contact with RAMP1
F27	sc[2] VDW/HP[3] to D94sc	
T30	mc[1] N-H H-bond donate D94sc	
	mc C=O H-bond accept N128sc	
	sc H-bond donate D94sc	
	sc VDW/HP to W72sc, F92sc, F95sc	
V32	sc VDW/HP to F95sc, W72sc, Y124sc	
G33	mc C=O H-bond accept W121sc	
P34	mc C=O H-bond accept S117sc	
	sc VDW/HP to H114sc, A116sc	
F35	mc C=O H-bond accept R119sc	
	sc VDW/HP to S117sc	
F37	sc VDW/HP to W72sc, G71mc	sc VDW/HP to W84mc C=O and W84sc
		sc minor VDW/HP to P85sc
Amide	C=O H-bond accept T122mc N-H	
	NH_2 H-bond donate T122mc C=O	

[1]mc=main chain
[2]sc=side chain
[3]VDW/HP=van der Waals/hydrophobic contacts

Table S2 (related to Fig 2). Summary of contacts between AM and CLR:RAMP2 ECD.

AM residue	Contact with CLR	Contact with RAMP2
K38	mc[1] C=O H-bond accept Q93sc[2]	
D39	mc N-H H-bond donate T37sc	
	mc C=O H-bond accept Q93mc N-H	
N40	mc C=O H-bond accept D94mc N-H	
A42	mc N-H H-bond donate D94sc	
	sc VDW/HP[3] to F95sc, F92sc, W72sc	
P43	sc VDW/HP to F92sc, W72sc	
K46	sc VDW/HP to W72sc	sc H-bond donate E105sc
		sc H-bond donate E101sc
I47	sc VDW/HP to W72sc, F95sc, Y124sc	
S48	mc C=O H-bond accept W121sc	
P49	mc C=O H-bond accept S117sc	
	sc VDW/HP to H114sc, A116sc	
Q50	mc C=O H-bond accept R119sc	
Y52	sc VDW/HP to W72sc, G71mc	sc H-bond donate E101sc
		sc H-bond accept R97sc
		sc minor VDW/HP to F111mc/sc, P112sc
Amide	C=O H-bond accept T122mc N-H	
	NH_2 H-bond donate T122mc C=O	

[1]mc=main chain
[2]sc=side chain
[3]VDW/HP=van der Waals/hydrophobic contacts

Table S3 (related to Fig 4). Summary of receptor function when stimulated with hαCGRP (pEC_{50} and E_{max} values) and cell surface expression for mutants of CLR in the CGRP receptor.

	cAMP				Surface Expression
**Mutant**	Wildtype (pEC_{50})	Mutant (pEC_{50})	Fold[4] change	E_{MAX} (% WT)	HA-CLR (% WT)
T37A[1]	9.15 ± 0.17 (5)	10.08 ± 0.23* (5)	8.5 (↑)	129.1 ± 12.6	108.5 ± 8.6
W69A[2]	9.44 ± 0.18 (7)	<6 (7)	>1000	Undetectable (7)	84.0 ± 6.1 (7)
D70A[2]	9.55 ± 0.18 (7)	<6 (7)	>1000	Undetectable (7)	83.6 ± 5.1 (7)
W72A[2]	9.93 ± 0.19 (6)	8.29 ± 0.29* (6)	44	76.6 ± 3.6* (6)	96.0 ± 8.4 (7)
D90A[2]	10.00 ± 0.03 (4)	9.50 ± 0.10** (4)	3	116.2 ± 4.1 (4)	103.9 ± 3.5 (3)
Y91A[2]	9.94 ± 0.09 (4)	8.30 ± 0.17* (4)	44	101.8 ± 9.9 (4)	93.9 ± 2.1 (2)
F92A[2]	9.88 ± 0.10 (5)	8.28 ± 0.07** (5)	40	94.9 ± 9.2 (5)	103.5 ± 1.1 (3)
Q93A[3]	9.33 ± 0.07 (4)	9.34 ± 0.07 (4)	-	112.9 ± 24.8 (4)	108.7 ± 3.7(4)
D94A[2]	9.80 ± 0.10 (5)	<6 (5)	>1000	Undetectable (5)	107.8 ± 7.6 (3)
F95A[2]	9.86 ± 0.17 (5)	7.59 ± 0.29* (5)	186	69.8 ± 8.6* (5)	111.4 ± 2.3 (3)
K103A[2]	9.91 ± 0.14 (4)	8.11 ± 0.19* (4)	63	97.6 ± 11.1 (4)	109.5 ± 3.7 (3)
H114A[3]	9.44 ± 0.09 (6)	8.12 ± 0.13 (6)***	21	85.8 ± 10.4 (6)	109.3 ± 17 (4)
S117A[3]	9.34 ± 0.06 (3)	8.92 ± 0.14 (3)*	3	90.4 ± 22.8 (3)	104.5 ± 7.3 (4)
R119A[2]	9.91 ± 0.14 (4)	8.16 ± 0.20* (4)	56	95.0 ± 6.8 (4)	107.1 ± 6.8 (6)
W121A[3]	9.56 ± 0.12 (3)	<6 (3)	>1000	Undetectable (3)	98.9 ± 10 (4)
T122A[3]	9.23 ± 0.06 (10)	7.93 ± 0.10 (10)***	20	103.0 ± 14.9 (10)	105.4 ± 4.3 (4)
Y124A[2]	9.76 ± 0.09 (5)	7.85 ± 0.09** (5)	81	79.6 ± 2.5* (5)	112.9 ± 4.6 (3)

[1]Data taken from Mapping interaction sites within the N-terminus of the calcitonin gene-related peptide receptor; the role of residues 23-60 of the calcitonin receptor-like receptor. Barwell J, Miller PS, Donnelly D, Poyner DR. Peptides. 2010 Jan;31(1):170-6

[2]These experiments were conducted using the ^{3}H cAMP method. An additional set of experiments using two mutants with the AlphaScreen cAMP method as per the AM_1 receptor gave mean pEC_{50}s of WT 9.57 ± 0.11 (3) and D94A <6 (3); WT 9.03 ± 0.06 (6) and W72A 7.03 ± 0.14*** (6).

[3]These experiments were conducted using the AlphaScreen cAMP method as per the AM_1 receptor. In a fourth experiment, W121A gave a pEC_{50} of 8.07 with a WT pEC_{50} of 9.36.

[4]Unless otherwise stated, values are fold-decreases.

For cAMP (pEC_{50}) data are the combined mean ± SEM (individual experiments). * $p < 0.05$; ** $p < 0.01$; *** $p < 0.001$ versus wild type receptor by unpaired t-test. For E_{MAX} and surface expression data are the combined mean ± SEM (individual experiments). * $p < 0.05$; ** $p < 0.01$; versus wild type receptor by one-way ANOVA, followed by Dunnett's post-hoc test.

Table S4 (related to Fig 5). Summary of receptor function when stimulated with hAM (pEC_{50} and E_{max} values) and cell surface expression for mutants of CLR or RAMP2 in the AM_1 receptor.

	cAMP				Surface Expression
**Mutant**	Wildtype (pEC_{50})	Mutant (pEC_{50})	Fold change	E_{MAX} (% WT)	HA-CLR (% WT)
**Calcitonin receptor-like receptor (CLR)**					
T37A	9.08±0.11 (3)	8.58±0.21 (3)	3	96.5±5.6 (3)	87.3±13.6 (4)
W72A	9.23±0.12 (4)	7.13±0.12 (4)***	126	35.5±11.4 (4)***	86.4±10.2 (4)
F92A	9.24±0.09 (3)	7.58±0.21 (3)**	46	42.8±2.1 (3)**	96.9±12.9 (4)
Q93A	9.25±0.10 (3)	9.20±0.12 (3)	-	111.7±14.5 (3)	99.5±2.7 (4)
D94A	9.13±0.15 (3)	8.20±0.14 (3)*	9	78.3±14.0 (3)	98.4±23.2 (4)
F95A	9.15±0.10 (3)	7.40±0.32 (3)**	56	73.7±6.2 (3)	111.8±18.4 (4)
H114A	9.07±0.03 (3)	8.28±0.25 (3)*	6	64.0±12.2 (3)	90.9±19.5 (4)
S117A	9.18±0.12 (3)	9.09±0.19 (3)	-	80.5±5.3 (3)	99.4±26.9 (4)
R119A	9.26±0.08 (4)	8.70±0.10 (4)**	4	84.9±8.3 (4)	102.5±24.3 (4)
W121A	9.32±0.10 (3)	6.99±0.05 (3)***	214	30.4±1.5 (3)***	102.2±15.3 (4)
T122A	9.31±0.18 (3)	8.41±0.15 (3)*	8	49.4±5.2 (4)**	106.0±15.7 (4)
Y124A	9.44±0.12 (3)	<6	>1000	Undetectable (3)	62.9±7.67 (4)**
**Receptor activity-modifying protein 2 (RAMP2)**					
L106R	9.13±0.15 (3)	9.28±0.20 (3)	-	91.4±7.9 (3)	121.7±24.9 (4)
R97A	9.25±0.10 (3)	9.16±0.11 (3)	-	82.9±1.0 (3)	85.0±5.2 (4)
E101A	9.26±0.09 (6)	7.84±0.09 (6)***	26	90.4±9.37 (6)	100.8±4.39 (8)
E105A	9.05±0.07 (3)	9.08±0.09 (3)	-	91.7±6.5 (3)	103.1±4.3 (4)
E101A/ R97A	9.44±0.12 (3)	8.07±0.03 (3)***	23	50.4±8.90 (3)	85.7±7.56 (4)
E101A/ E105A	9.44±0.12 (3)	7.70±0.06 (3)***	55	46.3±4.90 (3)	75.3±8.32 (4)

For cAMP (pEC_{50}) data are the combined mean ± SEM (individual experiments). * $p < 0.05$; ** $p < 0.01$; *** $p < 0.001$ versus wild type receptor by unpaired t-test. For E_{MAX} and surface expression data are the combined mean ± SEM (individual experiments). ** $p < 0.01$; *** $p < 0.001$ versus wild type receptor by one-way ANOVA, followed by Dunnett's post-hoc test.

Supplemental Experimental Procedures

Plasmid construction and protein production and characterization. Bacterial pETDuet1 expression plasmids encoding tethered MBP-hRAMP1.24-111-(Gly-Ser-Ala)$_3$-hCLR.29-144-H$_6$ and MBP-hRAMP2.55-140-(Gly-Ser-Ala)$_3$-hCLR.29-144-H$_6$ (amino acid numbers indicated) fusion proteins co-expressed with DsbC were constructed using the Gibson Assembly cloning method with Gibson Assembly master mix (New England Biolabs). Plasmids for expression of tagged full-length CLR and RAMP1 or -2 receptors in mammalian cells were previously described (Barwell et al., 2010; Watkins et al., 2014). Site directed mutagenesis was performed with the QuikChange II kit (Agilent) or using the Gibson Assembly method. Primer sequences are available upon request. All constructs were verified by automated DNA sequencing.

The tethered ECD fusion proteins were expressed in *E. coli*, purified, and characterized for peptide binding with an AlphaScreen peptide binding assay as previously described (Moad and Pioszak, 2013), except that the AlphaScreen competition assays also included 0.3% (v/v) Triton X-100 in the reaction buffer. Triton X-100 minimized, but did not completely prevent, apparent aggregation of the CGRP(27-37)NH_2 [D31, P34, F35] peptide that was surprisingly observed only with the MBP-hRAMP1.24-111-(Gly-Ser-Ala)$_3$-hCLR.29-144-H$_6$ protein and not with MBP-hRAMP1.24-111-(Gly-Ser)$_5$-hCLR.29-144-H$_6$ or MBP-hRAMP2.55-140 [L106R]-(Gly-Ser-Ala)$_3$-hCLR.29-144-H$_6$ proteins (data not shown). The apparent aggregation of the CGRP analog prevented it from fully competing the binding signal to background levels and hence the reported IC_{50} values for the CGRP analog peptides binding to the CGRP receptor crystallization construct are likely a bit higher than the true values. Competitor

peptide concentrations higher than 200 μM were avoided because some of the peptides began to exhibit non-specific inhibition at concentrations > 200 μM as assessed in control reactions in which the donor and acceptor beads were brought together by a Biotin-$(Gly)_6$-$(His)_6$ peptide. The binding experiments were conducted at least three times with each independent experiment performed with duplicate samples. pIC_{50} values are stated as the mean of the replicate independent experiments ± S.E.M. Although slight variation in pIC_{50} values for a given peptide in assays conducted on different days was occasionally observed, the rank order of IC_{50} values for the various peptides and the magnitude of their differences were very reproducible.

Peptides. Custom synthetic peptides for binding studies and crystallization were from RS Synthesis (Louisville, Kentucky) other than the CGRP(27-37)NH_2 [D31, P34, F35] peptide used for crystallization, which was assembled by Fmoc SPPS on Rink amide polystyrene resin using a Tribute synthesizer (Protein Technologies, Tucson, Az) with 20% (v/v) piperidine in DMF as Fmoc deblocking reagent (2 x 5 mins) and HATU/DIPEA (20 mins) as coupling reagents. The peptide was cleaved from the resin with concomitant removal of side chain protecting groups with 95% TFA/2.5% TIPS/2.5% water (v/v/v) for 2 h and recovered by precipitation into cold diethyl ether and isolated by centrifugation (221 mg). Purification of a portion (110 mg) by RP-HPLC on a C18 column (Waters Xterra, 19 x 300 mm) afforded the title compound (27.4 mg, >95% purity by HPLC), observed mass (ESI+) $(M+H)^{1+}$ = 1195.0, calculated mass 1196.4. For cell-based assays Human AM(1-52) was from Bachem and hαCGRP(1-37) was synthesized in-house (PWH) or was from Bachem.

Crystallization, data collection, structure determination, and analysis. The tethered MBP-RAMP1 ECD-CLR ECD and MBP-RAMP2 ECD [L106R]-CLR ECD fusion proteins were incubated for 1 h on ice in the presence of CGRP(27-37)NH_2 [D31, P34, F35] or AM(25-52)NH_2 (1:1.3 protein:peptide molar ratio), respectively, in 10 mM Tris-HCl, pH 7.5, 50 mM NaCl, 1 mM EDTA, 1mM maltose and spin concentrated to 30 mg/ml for crystallization. Crystals were grown by the hanging drop vapor diffusion method at 20 °C with a reservoir solution of 22% PEG3350, 8% Tacsimate (Hampton Research), pH 6.0 for the CGRP receptor complex or 19% PEG3350, 0.1 M Tris-HCl, pH 8.3, 225 mM sodium acetate, and 20% ethylene glycol for the AM_1 receptor complex. Microseeding was used to obtain the best CGRP receptor complex crystals for data collection. CGRP receptor complex crystals were cryoprotected by dialysis to mother liquor solution containing 12% PEG400; AM_1 receptor complex crystals were suitably cryoprotected in their growth condition. Crystals were flash frozen in liquid nitrogen and diffraction data were collected remotely at beamline 21-ID-G (λ = 0.97857 Å) of the Advanced Photon Source (Argonne, IL). Data from single crystals were indexed, integrated, and scaled with HKL2000 v. 705b (Otwinowski and Minor, 1997) and further processed/analyzed with the CCP4 suite v.6.4.0 (Winn et al., 2011) in preparation for molecular replacement (MR). The structures were solved with Phaser v. 2.5.6 (McCoy et al., 2007) using an MBP search model with maltose removed (PDB 3C4M) followed by ligand-free CLR:RAMP1 ECD (PDB 3N7S) or CLR:RAMP2 ECD heterodimer search models (PDB 3AQF). The MR solutions were rigid body refined with REFMAC5 v. 5.8.0073 (Murshudov et al., 1997) treating MBP, CLR, and RAMP1 or RAMP2 as separate rigid bodies. At this stage $2mF_o\text{-}DF_c$ and $mF_o\text{-}DF_c$ electron density maps clearly

showed bound maltose and CGRPmut or AM peptide. The models were completed by iterative rounds of manual rebuilding in COOT (Emsley et al., 2010) and TLS and restrained refinement with REFMAC5. NCS restraints were applied to the three molecules in the ASU of the CGRP receptor complex structure with the restraints relaxed for areas where the molecules differed. Structure analysis used PyMol (Schrodinger) and programs in the CCP4 suite and figures were prepared with PyMol. Structural superpositions were performed with the PyMol align command (for Cα atoms) utilizing outlier rejection.

Homology Modeling. Homology models of RAMP3 were generated using Modeller 9v12 (Sali and Blundell, 1993). CGRPmut-bound CLR:RAMP1 and AM-bound CLR:RAMP2 template structures were used either singularly or in combination to generate the models. 6000 models were generated, refined using Rosetta 3.5 (Rohl et al., 2004) and ranked using the OPUS_PSP scoring function (Lu et al., 2008). The 600 best scoring structures were then clustered into 0.1nm bins using the g_cluster function as implemented in Gromacs (Pronk et al., 2013). The best scoring structure from the largest, best scoring cluster was then selected.

Supplemental References

Barwell, J., Miller, P.S., Donnelly, D., and Poyner, D.R. (2010). Mapping interaction sites within the N-terminus of the calcitonin gene-related peptide receptor; the role of residues 23-60 of the calcitonin receptor-like receptor. Peptides *31*, 170-176.

Emsley, P., Lohkamp, B., Scott, W.G., and Cowtan, K. (2010). Features and development of Coot. Acta Crystallogr D Biol Crystallogr *66*, 486-501.

Lu, M., Dousis, A.D., and Ma, J. (2008). OPUS-PSP: an orientation-dependent statistical all-atom potential derived from side-chain packing. J Mol Biol *376*, 288-301.

McCoy, A.J., Grosse-Kunstleve, R.W., Adams, P.D., Winn, M.D., Storoni, L.C., and Read, R.J. (2007). Phaser crystallographic software. J Appl Crystallogr *40*, 658-674.

Moad, H.E., and Pioszak, A.A. (2013). Selective CGRP and adrenomedullin peptide binding by tethered RAMP-calcitonin receptor-like receptor extracellular domain fusion proteins. Protein Sci *22*, 1775-1785.

Murshudov, G.N., Vagin, A.A., and Dodson, E.J. (1997). Refinement of macromolecular structures by the maximum-likelihood method. Acta Crystallogr D Biol Crystallogr *53*, 240-255.

Otwinowski, Z., and Minor, W. (1997). Processing of X-ray diffraction data collected in oscillation mode. In Methods in Enzymology, C.W.J. Carter, and R.M. Sweet, eds. (New York: Academic Press), pp. 307-326.

Pronk, S., Pall, S., Schulz, R., Larsson, P., Bjelkmar, P., Apostolov, R., Shirts, M.R., Smith, J.C., Kasson, P.M., van der Spoel, D*., et al.* (2013). GROMACS 4.5: a high-throughput and highly parallel open source molecular simulation toolkit. Bioinformatics *29*, 845-854.

Rohl, C.A., Strauss, C.E., Misura, K.M., and Baker, D. (2004). Protein structure prediction using Rosetta. Methods Enzymol *383*, 66-93.

Sali, A., and Blundell, T.L. (1993). Comparative protein modelling by satisfaction of spatial restraints. J Mol Biol *234*, 779-815.

Watkins, H.A., Walker, C.S., Ly, K.N., Bailey, R.J., Barwell, J., Poyner, D.R., and Hay, D.L. (2014). Receptor activity-modifying protein-dependent effects of mutations in the calcitonin receptor-like receptor: implications for adrenomedullin and calcitonin gene-related peptide pharmacology. Br J Pharmacol *171*, 772-788.

Winn, M.D., Ballard, C.C., Cowtan, K.D., Dodson, E.J., Emsley, P., Evans, P.R., Keegan, R.M., Krissinel, E.B., Leslie, A.G., McCoy, A*., et al.* (2011). Overview of the CCP4 suite and current developments. Acta Crystallogr D Biol Crystallogr *67*, 235-242.
